# Selected Ion Monitoring for Orbitrap-Based Metabolomics

**DOI:** 10.3390/metabo14040184

**Published:** 2024-03-25

**Authors:** Wenyun Lu, Matthew J. McBride, Won Dong Lee, Xi Xing, Xincheng Xu, Xi Li, Anna M. Oschmann, Yihui Shen, Caroline Bartman, Joshua D. Rabinowitz

**Affiliations:** 1 Lewis Sigler Institute for Integrative Genomics, Princeton University, Princeton, NJ 08544, USA; wlu@princeton.edu (W.L.);; 2 Department of Chemistry, Princeton University, Princeton, NJ 08544, USA; 3 DOE Center for Advanced Bioenergy and Bioproducts Innovation, Princeton University, Princeton, NJ 08544, USA; 4 Department of Chemical Biology, Ernest Mario School of Pharmacy, Rutgers University, Piscataway, NJ 08854, USA; 5 Department of Bioengineering, University of Pennsylvania, Philadelphia, PA 19104, USA; 6 Department of Pharmacology, University of Pennsylvania, Philadelphia, PA 19104, USA; 7 Rutgers Cancer Institute of New Jersey (CINJ), Rutgers University, New Brunswick, NJ 08901, USA; 8 Ludwig Institute for Cancer Research, Princeton University, Princeton, NJ 08544, USA

**Keywords:** selected ion monitoring, SIM, full scan, orbitrap, metabolomics, fluxomics, isotope tracing, isotope labeling, signal-to-noise ratio, relative standard deviation

## Abstract

Orbitrap mass spectrometry in full scan mode enables the simultaneous detection of hundreds of metabolites and their isotope-labeled forms. Yet, sensitivity remains limiting for many metabolites, including low-concentration species, poor ionizers, and low-fractional-abundance isotope-labeled forms in isotope-tracing studies. Here, we explore selected ion monitoring (SIM) as a means of sensitivity enhancement. The analytes of interest are enriched in the orbitrap analyzer by using the quadrupole as a mass filter to select particular ions. In tissue extracts, SIM significantly enhances the detection of ions of low intensity, as indicated by improved signal-to-noise (S/N) ratios and measurement precision. In addition, SIM improves the accuracy of isotope-ratio measurements. SIM, however, must be deployed with care, as excessive accumulation in the orbitrap of similar *m*/*z* ions can lead, via space-charge effects, to decreased performance (signal loss, mass shift, and ion coalescence). Ion accumulation can be controlled by adjusting settings including injection time and target ion quantity. Overall, we suggest using a full scan to ensure broad metabolic coverage, in tandem with SIM, for the accurate quantitation of targeted low-intensity ions, and provide methods deploying this approach to enhance metabolome coverage.

## 1. Introduction

The quantitative analysis of small molecule metabolites in biological samples presents an analytical challenge [1,2,3]. Metabolites have both diverse physical and chemical properties and exist across wide concentration ranges. Serum metabolite concentrations range from mM to pM [4]. The quantitation of pre-determined (targeted) sets of metabolites (including targeted labeled forms) can be achieved through liquid chromatography coupled with triple quadrupole (LC-QqQ) mass spectrometry [5,6,7]. Liquid chromatography high-resolution mass spectrometry (LC-HRMS) provides an alternative approach that can both detect known metabolites (with their isotope-labeled forms) and unexpected or novel metabolites [8,9]. Available commercial instruments include Quadrupole Time-of-Flight (QTOF) systems and Quadrupole Orbitrap instruments [10,11]. Analyses are typically performed using full scan with metabolite identification using the accurate mass at the MS1 level and/or from the MS2 spectrum, paired with known retention time on the LC column established using authentic standards [12,13,14]. Ion signals are converted to absolute concentrations through calibration with standards, either unlabeled or isotopically labeled [2,15,16]. To evaluate the quantitative performance, the two most relevant criteria are:

(1) Signal-to-noise ratio (S/N), where the noise refers to the electronic noise from the instrument detector [17]. A high S/N ratio correlates with better data quality;
(2) Relative standard deviation (RSD) from multiple measurements of the same sample [18]. A low RSD implies better precision.

The greatest quantitation challenge arises with ions of low intensity. Notably, low-intensity ions include metabolites of great biological importance, such as many glycolytic intermediates. Isotope-tracing studies using in vivo animal models bring additional challenges, as metabolite-labeling fractions are typically in the range of 1–25% [19,20]. Thus, there is a practical need to perform accurate quantitation for those ions of low intensity including isotope-labeled forms.

Quantitation performance can be improved in different ways, depending on the type of instrument. QTOF instruments have very high scan rates with each transient taking about 1 microsecond. The data are typically averaged over many thousands of transients, improving S/N ratio [11]. Such an approach is not suitable for an Orbitrap instrument, as the Orbitrap has a comparatively slow scan rate (e.g., 128 ms for one scan at 60 K mass resolution on Exploris 480 instrument).

An alternative approach is to use selected ion monitoring (SIM), where the quadrupole operates as a narrow mass filter, removing ions outside the specified scan range [21]. Ions within the scan range are allowed to accumulate in a storage device before being sent to orbitrap for analysis. To date, the literature lacks a comprehensive evaluation of the utility of SIM for metabolomics. Here, we systematically investigate the quantitative performance of full scan versus SIM, using a range of sample types, including isotope-labeled standards spiked in biological samples, and biological samples with or without isotope tracers. We show that SIM improves quantitative performance. This is largely achieved through longer injection time so that, compared to full scan, more of the targeted ions are available for detection. On the other hand, the increased injection of ions in a narrow *m*/*z* window can result in space charge effect-induced signal loss and ion coalescence when too many ions of close *m*/*z* are present inside the orbitrap. This effect can be minimized by optimizing scan-parameter settings, by adjusting the targeted number of ions to inject the orbitrap, i.e., automatic gain control (AGC) target, and the maximum injection time (IT_max_). Methods that blend full scan and SIM to increase the breadth and accuracy of metabolome quantitation are provided.

## 2. Materials and Methods

### 2.1. Metabolite Extraction from Yeast I. orientalis

Wild type *I. orientalis* SD108, a yeast strain of industrial utility for organic acid production [22], was grown in a shaker at 250 rpm at 30 °C in medium containing 20 g/L glucose (Sigma-Aldrich, St. Louis, MI, USA, D9434) and 6.7 g/L yeast nitrogen base (YNB) without amino acids (Sigma-Aldrich, Y0626). The metabolism was quenched and the metabolites were extracted when cultures reached exponential phase at OD_600_ = 1 [23]. In total, 2.4 mL yeast cultures were quickly vacuum filtered onto a nylon membrane filter (0.45 µm, Millipore), and immediately submerged in 1.5 mL acetonitrile:methanol:water (40:40:20) with 0.5% formic acid, precooled at −20 °C. After 10 min, 132 μL of 15.8 g/L NH_4_HCO_3_ solution was added to neutralize the formic acid. The mixture of cell debris and extraction solvent was transferred to a 1.5 mL Eppendorf tube and centrifuged at 14,000 rpm at 4 °C for 20 min, and the supernatant was collected for analysis. 

### 2.2. Metabolite Extraction from Mouse Tissues and Tumors

Frozen tissue or tumor samples were first weighed (~20 mg each) and transferred to 2.0 mL Eppendorf tubes on dry ice. The samples were then ground into powder with a cryomill machine (Retsch, Newtown, PA, USA) maintained at a cold temperature using liquid nitrogen. Thereafter, for every 25 mg tissue (now in the form of a powder), 1 mL 40:40:20 acetonitrile:methanol:water with 0.5% formic acid was added to the tube, vortexed, and allowed to sit on ice for 10 min [24], and 85 μL 15% NH_4_HCO_3_ (*w*:*v*) was added and vortexed to neutralize the samples. The samples were incubated on ice for another 10 min and then centrifuged at 14,000 rpm for 25 min at 4 °C. The supernatants were transferred to another Eppendorf tube and centrifuged at 14,000 rpm again for 25 min at 4 °C with the supernatant collected for analysis.

### 2.3. Liquid Chromatography-Mass Spectrometry (LC-MS)

The LC-MS analysis was performed on a Vanquish UHPLC system coupled with an Exploris 480 orbitrap mass spectrometer. LC separation was achieved using a Waters XBridge BEH Amide column (2.1 × 150 mm, 2.5 µm particle size) with a 25 min gradient (Figure S1) [23]. The retention times of ~600 metabolites using authentic standards are provided in Table S1. The Exploris 480 mass spectrometer was operated in full scan mode and/or SIM mode at MS1 level with the target analytes detected using an accurate mass within a 5 ppm window, with a resolving power of 120 K at *m*/*z* 200. Unless otherwise noted, the typical scan parameters for full scan are as follows: scan range *m*/*z* 120–1000 (positive mode) and *m*/*z* 70–1000 (negative mode), AGC target 1 × 10^7^, and IT_max_ 200 ms. The typical SIM parameters are the following: scan range ±1.5 Da of the ion of interest, AGC target of 1 × 10^6^, and IT_max_ of 200 ms. The other instrument parameters are the following: spray voltage 3200/2800 V (positive/negative mode), sheath gas 35 (Arb), aux gas 10 (Arb), sweep gas 0.5 (Arb), ion transfer tube temperature 300 °C, vaporizer temperature 35 °C, internal mass calibration on, and RF lens 60%. A set of experiments was carried out to evaluate full scan and SIM performance:

i. Evaluating the signal-to-noise (S/N) ratios of isotope-labeled standards spiked into a mouse-liver extract.

A mouse-liver extract was prepared as above, and six isotope-labeled standards were spiked in at two concentrations differing by 1000-fold (Table S2). The samples were analyzed in either full scan or SIM in negative mode.

ii. Evaluating the relative standard deviation for ions of low intensity

*I. orientalis* extract and mouse kidney extract were evaluated by a full scan to identify 10 metabolite ions of low intensity in positive and 10 in negative mode. The samples were then run five times in the SIM mode and separately five times in full scan mode with two different AGC target settings, a high AGC target (1 × 10^7^) and a low AGC target (1 × 10^6^) (Table S3). RSDs were determined.

iii. Determination of isotope ratios with or without isotope tracers

Mouse quadriceps muscles were collected from male wild-type C57/BL6 mice (12 weeks old) that either did or did not receive an infusion of 400 mM [U-^13^C] glucose at a rate of 0.1 μL per minute per gram body weight for 3 h and were flash-frozen in liquid nitrogen [19]. Extracts were then prepared as above, and analyzed in negative mode focusing on the three glycolysis intermediates: 3-phosphoglycerate (3PG), hexose 6-phosphate (HxP), and fructose 1,6-bisphosphate (FBP) (Table S4). The SIM parameters are (with varying IT_max_ to test the importance of this parameter) the following:

3PG: *m*/*z* 183–190, AGC target 1 × 10^6^, IT_max_ 50, 100, 200, 300, 500, or 1000 ms;

FBP: *m*/*z* 338–346, AGC target 1 × 10^6^, IT_max_ 50, 100, 200, 300, 500, or 1000 ms;

HxP: *m*/*z* 257–267, AGC target 1 × 10^6^, IT_max_ 5, 10, 20, 30, 50, or 100 ms.

iv. Evaluating ion coalescence with different settings for the AGC target and IT_max_

Mouse colorectal tumor extract after infusion with 8 nmol/min/g body weight of ^13^C-formate at 80 mM (see Supplementary Materials for details) for 13 h [19] was prepared as above, and the sample was analyzed in negative mode with a resolving power of 480 K at *m*/*z* 200, focusing on the detection of the labeled forms of adenosine triphosphate (ATP). The full scan parameters are the following: range *m*/*z* 70–1000, AGC target settings 1 × 10^7^, 7.5 × 10^6^, 5 × 10^6^, 2.5 × 10^6^, 5 × 10^5^, and 2 × 10^5^, and IT_max_ settings were 1000, 750, 500, 250, and 50 ms, for a total of 30 conditions. The SIM parameters are the following: Range *m*/*z* 504–510, AGC target 1 × 10^6^, 7.5 × 10^5^, 5 × 10^5^, 2.5 × 10^5^, 5 × 10^4^, and 2 × 10^4^, and IT_max_ settings were 1000, 750, 500, 250, and 50 ms, for a total of 30 conditions.

### 2.4. Data Analysis

Thermo raw data files were analyzed using either Qualbrowser or Freestyle to obtain the information of signal intensity (S), electronic noise (N), and injection time (IT) at the apex of the chromatogram for the ions of interest (see Supplementary Materials for details). The metabolites were identified using an accurate mass with a ±5 ppm window together with the retention time from the standards (Table S1). Alternatively, the raw data were converted to a mzxml format using ProteoWizard [25] and analyzed using El-Maven [26]. In all cases, the signal intensity from Thermo data files is the normalized signal in the unit of ion counts per second (cps):

S = (actual number of ions detected by orbitrap analyzer)/(injection time)

For example, a signal intensity of 1 × 10^6^ may correspond to 1 × 10^5^ ions in the orbitrap with IT of 100 ms (less noisy) or 1 × 10^3^ ions with IT of 1 ms (more noisy).

## 3. Results and Discussion

### 3.1. SIM Decreases Nominal Signal Intensity but Improves Signal-to-Noise Ratio

**Figure 1:**
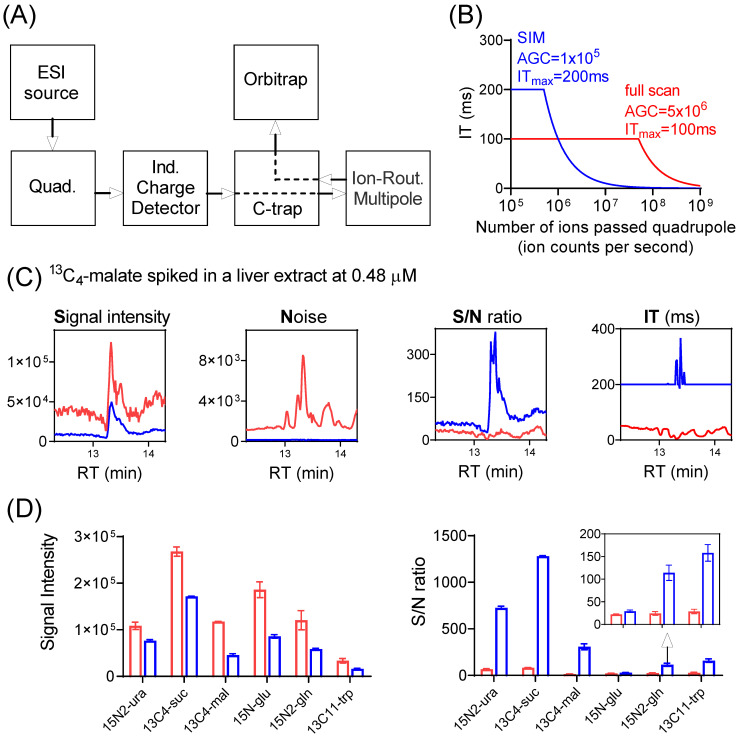
SIM improves signal-to-noise (S/N) ratio for low-abundance ions. (**A**) Major components of Exploris Orbitrap instrument. (**B**) Calculated ion-injection time (IT) as a function of the number of ions passed through the quadrupole (ion counts per second), for SIM with automatic gain control (AGC) target of 1 × 10^5^ and IT_max_ of 200 ms (blue trace), and full scan with AGC target of 5 × 10^6^ and IT_max_ of 100 ms (red trace). Note the IT is capped at IT_max_. (**C**) Nominal signal (ion counts per second, cps; note that actual ions detected = nominal signal x injection time), noise (N), signal-to-noise (S/N) ratio, and injection time (IT) for the detection of spiked ^13^C_4_-malate (*m*/*z* 137.0277 ± 5 ppm) in a mouse-liver extract using SIM (blue trace) and full scan (red trace). Note that when SIM scans were performed in multiplex mode, the actual IT for individual SIM may exceed the IT_max_ (Table S2). See Supplementary Materials for details. (**D**) Signal intensity (cps) and S/N ratio for the six isotope-labeled standards spiked into a mouse-liver extract at low concentrations (Table S2), using SIM (blue bar) and full scan (red bar), respectively.

A simplified schematic diagram showing major components of the Exploris 480 orbitrap mass spectrometer is depicted in Figure 1A [27,28]. Ions generated at the ion source are transferred to the quadrupole where either all ions pass through (full scan), or only ions within a selected *m*/*z* range pass through (SIM). Ions then reach the C-trap and enter the Ion-Routing Multipole (IRM), where ions are allowed to accumulate until the user-specified AGC target or IT_max_ is reached, whichever comes first. The ions are then brought back to C-trap and sent to Orbitrap for analysis. The control of the ion injection is achieved through the independent charge detector, which measures the number of ions passing through the quadrupole.

A full scan typically involves the rapid injection of ions with a broad *m*/*z* range that quickly reaches the AGC target and thus a brief injection time (Figure 1B). On the other hand, SIM involves fewer ions with a narrow *m*/*z* range being passed through the quadrupole and thus a longer injection time. Importantly, nominal signal is reported as detected ions divided by injection time. Thus, with a short injection time, even when only a small number of ions of a particular *m*/*z* is actually detected, the nominal signal can be large. When the quadrupole operates with a narrow *m*/*z* window (i.e., for SIM), some ions are lost during the filtering step. Therefore, SIM decreases the nominal signal even for the targeted *m*/*z* (Figure S2). Nevertheless, SIM can improve signal-to-noise ratio (S/N), because the actual number of detected ions is greater (Figure 1C, Table S2, for the detection of ^13^C_4_-malate standard spiked into a mouse liver extract at 0.48 μM). With SIM, an improved S/N ratio was observed for all isotope-labeled standards spiked in at low concentrations (Figure 1D, Table S2). The magnitude of the S/N gain was at least four-fold, with one exception: ^15^N-glutamate. The lesser improvement for ^15^N-glutamate is due to its mass being only 1 amu higher than unlabeled glutamate, which is of high intensity and whose inclusion accordingly results in a short injection time also for the SIM scan (Figure S3). SIM has no apparent benefit for ions of high intensity (Figure S4, Table S2). Thus, SIM improves S/N ratio selectively for ions of low intensity.

### 3.2. SIM Enables a More Precise Quantitation of Low-Intensity Ions

**Figure 2:**
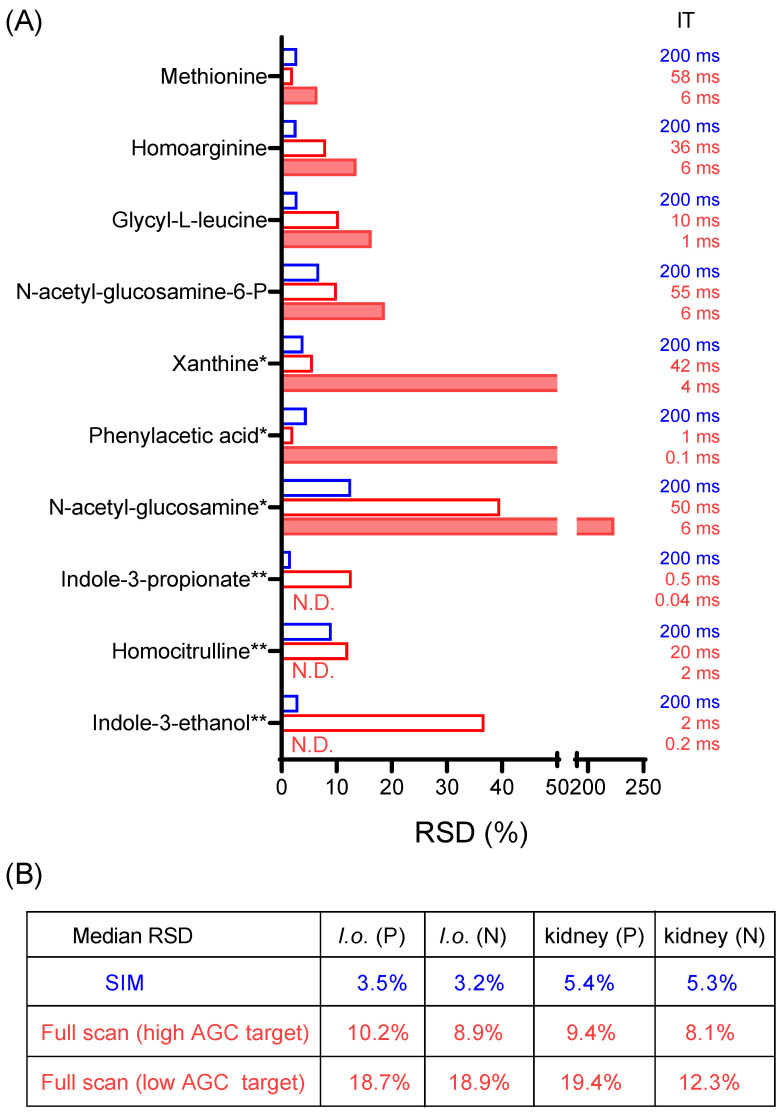
SIM enables detection and quantitation of low-intensity ions. (**A**) Relative standard deviation (RSD) for the signal intensity of 10 low-intensity metabolites from a yeast extract (*I. orientalis = I.o.*) in positive mode from five LC-MS runs of the same sample, using SIM or full scan with high AGC target of 1 × 10^7^ or full scan with low AGC target of 1 × 10^6^. *: metabolites detected in fewer than five measurements for full scan with low AGC target. **: metabolites not detected (N.D.) in any of the five measurements for full scan (low AGC target). (**B**) Summary of median RSD for 10 low-intensity metabolites for a yeast extract and a mouse-kidney extract, in positive (P) and negative mode (N), respectively. Data excludes those cases where RSD is not available (due to metabolites not being detected). Full data is presented in Table S3.

To get a sense of quantitative precision for the full scan and SIM from real biological samples, a yeast exact and a mouse-kidney extract were first analyzed in full scan mode. From the full scan data, ten metabolite ions in either positive mode or negative mode with the signal intensity in the range of 10^4^−10^5^ were randomly selected (Table S3). Samples were then run five times in SIM, full scan with a high AGC target, or full scan with a low AGC target. The resulting RSDs in positive mode for the yeast extract are shown in Figure 2A, and the median RSDs for all data are summarized in Figure 2B.

SIM consistently exhibited the lowest RSD, followed by the full scan with a high AGC target, while the full scan with a low AGC target exhibited the least favorable RSD. Notably, out of the ten metabolites from the yeast extract in positive mode, three of them remained undetectable in the full scan with a low AGC target (Figure 2A). In contrast, these metabolites were reliably detected in SIM mode, underscoring the significant enhancement in quantitative performance with SIM.

### 3.3. SIM Improves Isotope-Ratio Determination

While the measurement of metabolite concentration provides useful information for many applications such as biomarker discovery, isotope-tracer studies provide additional insights into metabolic activity [20,29,30,31]. Here, a labeled tracer (such as ^13^C_6_-glucose) is introduced to the biological system, and the labeled fractions of metabolites of interest are measured. For tracer studies in intact mammals, metabolite labeling is preferably kept low to minimally perturb endogenous metabolism, with biologically important labeling often being as low as a fraction of one percent [19,20]. Thus, high-sensitivity and good accuracy are both important.

**Figure 3:**
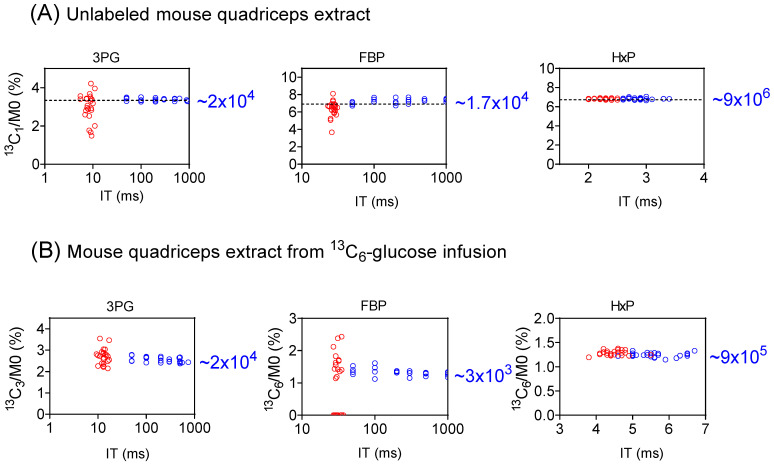
SIM improves isotope-ratio determination for low-intensity metabolite ions. (**A**) The measured isotope abundance for ^13^C_1_-3PG, -FBP, and -Hexose-P (HxP) from an unlabeled mouse-quadriceps extract, showing that SIM (blue) provides a more accurate isotope ratio for 3PG and FBP due to longer ion-injection time (IT), compared to full scan (red). HxP, an abundant ion, shows good data with either approach. The dashed horizontal line represents the true (calculated) isotope ratio arising from natural abundance. The number on the right side is the approximate signal intensity for the ^13^C_1_ form. (**B**) Similar improvements were seen for the ^13^C_3_-3PG and ^13^C_6_-FBP from mouse-quadriceps extract after ^13^C_6_-glucose infusion.

We evaluated the performance of full scan vs. SIM for the isotope-ratio determination for three glycolytic intermediates, 3-phosphoglycerate (3PG), hexose-phosphate (Hexose-P), and fructose-1,6-bisphosphate (FBP) using mouse quadricep-muscle extracts (Figure 3 and Figure S5). For the unlabeled extract (Figure 3A and Figure S5A), the labeling originates from the natural isotope abundance (^13^C_1_ and ^18^O_1_) [32]. Since the natural abundance is known, this provides a gold standard for measuring both the precision and accuracy of the isotopic forms [33]. As seen in Figure 3A, for 3PG, via a full scan, IT was 5 to 11 ms, and the ^13^C_1_/M0 ratios exhibited a relatively wide range, spanning from 1.5% to 4.2% relative to a true value of 3.34%. For SIM, IT increased to 50 to 937 ms, and the ratios narrowed down to an accurate range of 3.26% to 3.51%. Similar trends were observed for ^13^C_1_-FBP (Figure 3A), ^18^O_1_-3PG, and ^18^O_1_-FBP (Figure S5A). On the other hand, for Hexose-P, which is of a high intensity, the data quality in both the full scan and SIM was quite satisfactory (Figure 3A). Thus, SIM significantly enhances the determination of isotope ratios for low-intensity ions.

This observation is further supported from the analysis of a quadriceps extract following ^13^C_6_-glucose infusion, where the primary detected labeling species are ^13^C_3_-3PG, ^13^C_3_- and ^13^C_6_-FBP, ^13^C_3_- and ^13^C_6_-Hexose-P (Figure 3B and Figure S5B). For both 3PG and FBP, the precision of the isotope ratio measurement improved substantially from the full scan to SIM. For example, for FBP, the ^13^C_6_/M0 ratio ranged from 0 to 2.43%, with the ^13^C_6_ form not detected in 10 of 24 full scan runs (Figure 3B). In contrast, ^13^C_6_-FBP was readily detected in SIM with the ^13^C_6_/M0 ratio falling within a narrow range. On the other hand, data quality remained largely consistent between the full scan and SIM for Hexose-P, a high-intensity species. These results further highlight the advantages of SIM for the precise determination of isotope ratios for low-intensity ions.

### 3.4. Proper Setting of AGC Target and IT_max_ to Minimize Space-Charge Effect

Orbitrap is a type of trap instrument and thus is prone to the space-charge effect, in which too many ions inside the trap adversely affect the analytical performance [34]. One consequence of the space-charge effect is the signal loss when high numbers of ions of similar *m*/*z* are present inside the orbitrap. Examining the signal intensity of glutamine ion from a mouse-liver extract shows that the signal is similar with no apparent space-charge effect when IT is in the range of 0.3–10 ms, for both full scan and SIM (Figure S6, Table S5). Space-charge effect-induced signal loss starts to appear when IT is longer than 10 ms, and becomes more severe with a longer IT. Similarly, the signal intensity of the unlabeled peak of 3PG and FBP decreases with increasing IT in SIM mode (Figure S5). The signal loss due to the space-charge effect inside the orbitrap should not be confused with decreasing ionization efficiency at the electrospray ionization source due to co-eluting high abundance species, a phenomenon known as ion suppression [35,36].

**Figure 4:**
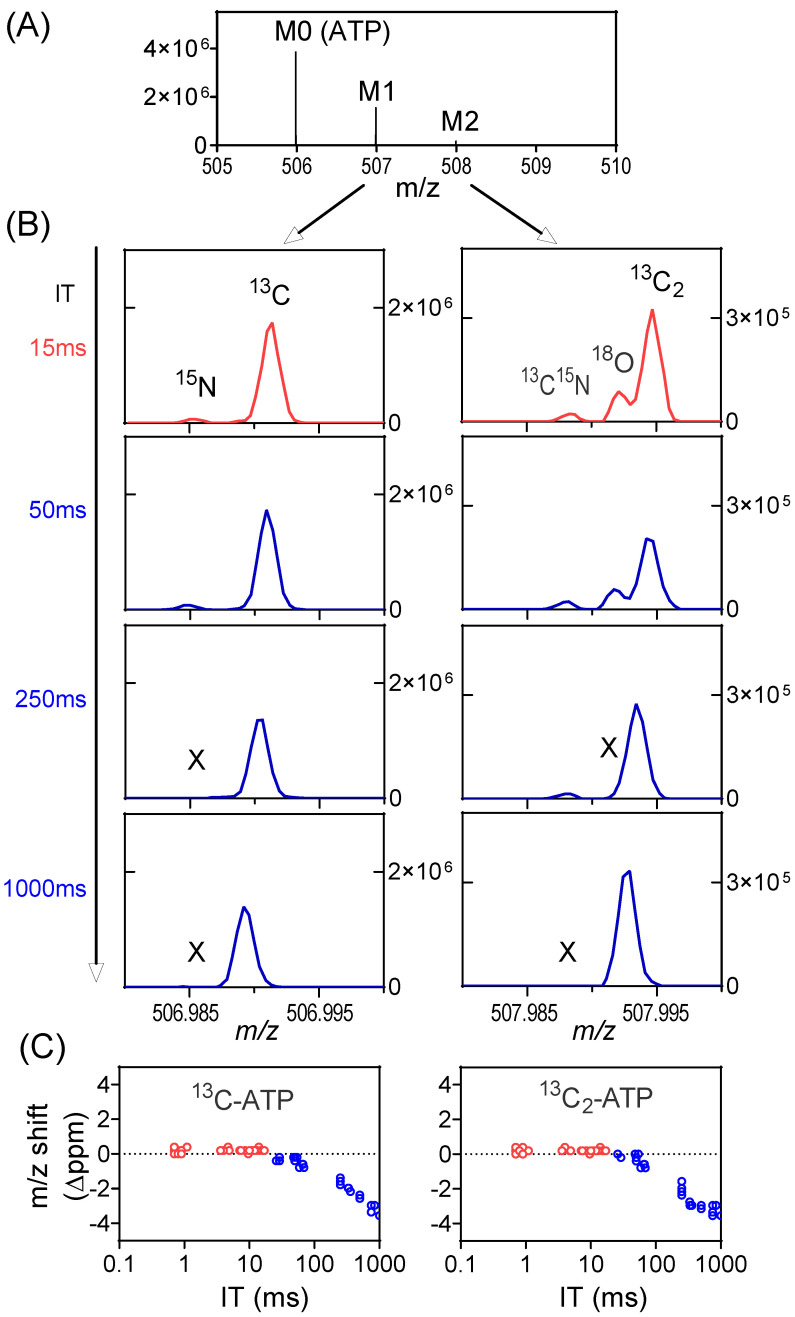
Space-charge effect and ion coalescence can be minimized by the proper setting of the AGC target and IT_max_. (**A**) Mass spectrum from a tumor extract with ^13^C-formate infusion at the retention time of ATP showing the unlabeled peak (M0), and M1 and M2 peaks. (**B**) Mass spectra showing the details of the M1 and M2 peak profiles in full scan (red trace) or SIM (blue trace) at different IT. All the isotope peaks were resolved in full scan and in SIM scans when IT is low. Some low abundant isotope peaks (e.g., ^15^N, ^18^O) go missing (marked as X) when IT is high, due to ion coalescence. In addition, *m*/*z* shift is also observed. (**C**) *m*/*z* shift (Δppm) of ^13^C-ATP and ^13^C_2_-ATP at different ITs in both full scan and SIM. The shift increases with IT in SIM scans due to ion coalescence.

The other consequence relates to the mass accuracy. With high numbers of ions in the orbitrap, the ion motion trajectory of individual ions is more likely to be affected by nearby ions (particularly those of the same *m*/*z* or similar *m*/*z*), resulting in worse mass accuracy. In the worst case, two ions with similar *m*/*z* may merge as a single peak on the mass spectrum, a phenomenon known as ion coalescence [37,38,39,40,41,42]. While SIM may improve signal-to-noise ratio, the high number of ions in a narrow *m*/*z* range also make it more prone to ion coalescence. As an example, we examined the labeling of ATP from a mouse tumor when infused with ^13^C-formate (Figure S7). The primary ions of interest are ^13^C_1_-ATP (with ^15^N-ATP nearby) and ^13^C_2_-ATP (with ^13^C^15^N-ATP and ^18^O-ATP nearby, Figure 4A). For the full scan (*m*/*z* 70–1000), the IT is brief (~15 ms) as the AGC target is quickly reached, and all ions of interest are resolved. They are also resolved for SIM when the IT is capped by the user at 50 ms (IT_max_ = 50 ms). When IT increases to 250 ms, ^15^N-ATP merges with ^13^C-ATP and ^18^O-ATP merges with ^13^C_2_-ATP (Figure 4B). When IT reaches 1000 ms, the ^13^C^15^N-ATP also merges with ^13^C_2_-ATP. Overall, there is a downward shift in the mass of ^13^C-ATP and ^13^C_2_-ATP with increasing IT in SIM as a result of ion coalescence (Figure 4C). The merging of multiple peaks with similar *m*/*z* will result in incorrect isotope-ratio measurements [43]. To obtain correct isotope enrichment, here, IT_max_ is set at 50 ms for SIM (Figure S8). The key message is that a longer injection time allows more ions to accumulate and increases the S/N ratio, but it can also result in increased risks of the space-charge effect and ion coalescence. Consequently, selecting the optimal IT_max_ value is important to strike a balance between sensitivity and accuracy.

### 3.5. Optimized Metabolome Quantitation by Combining Full Scan and SIM

Both full scan and SIM are MS1-based methods. Some considerations for their practical application in orbitrap LC-MS metabolomics are the following:

(1). A full scan with a high AGC target is effective for general metabolomics, especially for high-intensity species;
(2). SIM can be a valuable complement for low-intensity ions including isotope-labeled species;
(3). For measuring labeled forms, the SIM scan window should cover all masses of interest of that ion (e.g., from unlabeled to the highest labeled form);
(4). Full scan and SIM can be alternated within the same LC run;
(5). The orbitrap resolving power setting affects scan speed (Figure S9, Table S6). It is desirable to keep the resolving power low enough (i.e., scan fast enough) to maintain good chromatogram coverage (e.g., one data point per second).

To build an optimized method, a full scan using a high AGC target is a good starting point. Polarity switching allows for the collection of positive- and negative-mode full scan data in a single run. For isotopic-tracing studies focusing on central carbon metabolism, we often use a full scan followed by SIM scans targeting specific metabolites of interest and their labeled forms, as in the case of the ^13^C-labeling of mouse muscle (Figure 3). Timed SIM (tSIM) scans allow for many SIMs to be included in a single method, where SIMs are performed only around the expected retention time, similar to scheduled multiple reaction monitoring (MRM) on triple quadrupole instruments. Note that, in addition to knowing RT, MRM requires the *m*/*z* of the product ion and the optimal collision energy, while SIM does not [11]. Whether MRM or high-resolution SIM scanning yields more sensitive quantitation depends on the instrument and analyte, with an important factor being the analyte’s propensity to produce a single, characteristic, high-yield fragment (as required for MRM but not SIM). Multiplex SIM scans can potentially further increase the number of species to be included in a single run, where distinct ions are isolated separately, but accumulated in IRM and analyzed in orbitrap together. Many additional species can be captured by integrating high-resolution SIM with high-resolution full scans. Some scan methods that we routinely use in our laboratory are provided in Table 1.

**Figure 5:**
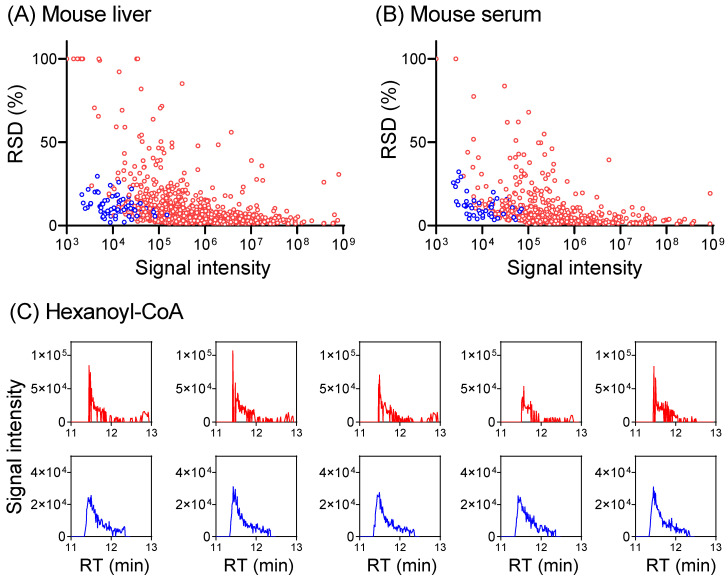
Combining SIM with a full scan provides more accurate metabolome quantitation. (**A**) Plot of RSD for 872 metabolites (>400 each in positive + negative mode, red dots) detected in full scan, and 75 metabolites in SIM (blue dots) from a mouse-liver extract. RSD increases with decreasing signal intensity in the full scan and SIM lowers RSD for low-intensity ions. Complete data are provided in Table S7. For metabolites with RSD >100%, RSD was plotted as 100% for visualization purposes. (**B**) Similar results for serum (482 total metabolites in full scan, positive + negative mode, red dots) with 49 SIM (blue dots). (**C**) Extracted ion chromatograms for hexanoyl-CoA (*m*/*z* 866.196 ± 10 ppm in positive mode, RT 11.45 min) in full scan (red trace) and SIM (blue trace) from five LC-MS runs of the same mouse-liver extract. The injection times are 1 ms for full scan and 30 ms for SIM at 11.45 min.

To develop a hybrid full scan–SIM method for enhanced metabolome quantitation, we first conducted a full scan analysis of a mouse serum extract and a mouse-liver extract to identify metabolite ions of low intensity. Knowing *m*/*z* and RT for these low-intensity ions, a timed SIM method was implemented in multiplexed mode to allow for the monitoring of as many SIMs as possible, together with a full scan in the same LC-MS run (Table S7). The data acquisition alternates between the full scan and targeted SIMs, with the full scan for general metabolomics, and SIMs for the quantitation of targeted low-intensity ions. For the liver extract in negative mode, 418 metabolites were quantified in full scan mode. The inclusion of the SIM scans improves the signal-to-noise ratio for additional 38 metabolites to permit their quantification, including ~10 CoA species that are poorly detected in full scan mode (Figure 5 and Figure S11, Table S7). Similar benefits were obtained also in positive mode and for serum. Thus, combining a full scan with targeted SIM scans together in a single LC-MS run enables the quantitation of more metabolites.

## 4. Conclusions

A high-resolution, accurate-mass full scan on orbitrap is routinely used for metabolomics, both for metabolite quantitation and unknown metabolite discovery. Its quantitative performance may not be optimal for all metabolites due to an insufficient number of ions being sent to the orbitrap. This is particularly the case for low-intensity ions when AGC targets are set low. We show here that quantitative performance can be improved by using SIM that filters ions within a pre-selected *m*/*z* range to accumulate before orbitrap analysis. SIM improves the S/N ratio and measurement precision and accuracy. On the other hand, too many ions of similar *m*/*z* can adversely affect the quantitative performance due to space-charge effect, and it is important to set a correct AGC target and IT_max_ to achieve the benefits of SIM without this pitfall. Effective SIM methods for serum and liver metabolomics are provided in Table S7.

## Figures and Tables

**Table 1:** Suggested scan parameter settings for selected applications using 25 min HILIC method for metabolomics *.

Analysis of Interest	Polarity	Scan Parameter Setting
General metabolomics	Polarity switching	Positive-mode full scan (*m*/*z* 59–1000) + negative-mode full scan (*m*/*z* 70–1000)
Deep metabolomics	Separate runs in positive mode and negative mode, respectively	Full scan + targeted SIMs in multiplex mode **
Central carbon metabolism, glycolysis with ^13^C labeling	Negative mode	Full scan (*m*/*z* 70–1000) + SIM for 3PG (*m*/*z* 184–190, RT 13–15 min) +SIM for FBP (*m*/*z* 337–347, RT 13–15 min)
Central carbon metabolism, NAD^+^, NADH, NADP^+^, NADPH	Polarity switching	Full scan (negative mode) + full scan (positive mode) + SIM (*m*/*z* 662–670, positive mode, RT 12–14.5 min) + SIM (*m*/*z* 772–780, positive mode, RT 13–15 min)
Central carbon metabolism for samples containing a high level of phosphate ***	Negative mode	Full scan (*m*/*z* 70–96) + full scan (*m*/*z* 98–194) + full scan (*m*/*z* 196–1000)
Central carbon metabolism, ^13^C-labeling of ATP	Negative mode	Full scan (*m*/*z* 70–1000) + SIM (*m*/*z* 505–515, RT 13–15 min, IT_max_ 50 ms, R = 480 K)

*: Unless otherwise noted, scan parameters for full scans are AGC target 1 × 10^7^, IT_max_ 100 ms, and R = 120 K at *m*/*z* 200. Scan parameters for SIM are AGC target 1 × 10^6^, IT_max_ 100 ms, and R = 120 K at *m*/*z* 200. **: See method in Supplementary Materials. ***: Phosphate typically due to incomplete removal of culture medium (Figure S10). The full scans are chosen to exclude phosphate anion.

## Data Availability

All relevant data are presented in the Supplementary Materials. Raw data files are deposited at https://massive.ucsd.edu/ with accession ID MSV000094084 (accessed on 1 March 2024).

## Supplementary Materials

The following supporting information can be downloaded at: https://www.mdpi.com/article/10.3390/metabo14040184/s1, Supporting Methods; S1. Chemicals and reagents; S2. Yeast culture condition; S3. Animal studies and tissue collection; S4. Animal infusions with ^13^C-glucose; S5. [1-^13^C]-2-deoxyglucose infusion and tissue collection; S6. [^13^C]-formate infusion for mouse colorectal tumor; S7. Metabolite extraction from serum; S8. Liquid chromatography; S9. Using Xcalibur Qual Browser to obtain information on noise (N) and injection time (IT); S10. Resolving power and scan speed on Exploris 480; S11. Natural isotope abundance calculation; S12. Setting up hybrid scan method covering both full scan and SIM on Exploris 480; Supporting Figures: Figure S1. Separation of selected metabolite isomers; Figure S2. Narrower scan width results in lower signal intensity; Figure S3. Inclusion of high intensity ions such as ^12^C-glu diminishes the benefits of SIM; Figure S4. SIM does not improve S/N ratio for high intensity ions; Figure S5. Additional data for isotope ratio determination from mouse quadriceps extracts; Figure S6. Space-charge caused signal drop and *m*/*z* shift for the glutamine ion from a liver extract when IT is long in SIM; Figure S7. Diagram showing the labeling positions of ATP from [^13^C]-formate; Figure S8. Additional data on ATP labeling from [^13^C]-formate infusion; Figure S9. Resolving power and scan speed on Exploris 480; Figure S10. The prominent peaks from phosphate at high concentrations from a T-cell extract; Figure S11. Detection of CoA metabolites using SIM vs. full scan from a mouse-liver extract; Figure S12. The interference peak of ^13^C_1_-2-DG6P from a mouse-colon extract; Figure S13. Examples of interference peaks from in-source fragments. III. Supporting Tables: Table S1. List of ~600 metabolite standards with RT (Excel table); Table S2. Additional data on SIM vs. full scan for six isotope-labeled standards spiked into a mouse-liver extract (Excel table); Table S3. Additional data on the detection of ten low-abundance metabolites from a mouse-kidney extract, and from an *I.o.* extract (Excel table); Table S4. Additional data on isotope-ratio determination from mouse-quadriceps extracts (Excel table); Table S5. Signal intensity and mass accuracy of glutamine ion from a mouse-liver extract in a full scan (*m*/*z* 70–1000) or SIM (*m*/*z* 144.5–145.5) under different AGC targets and IT_max_ settings (Excel table); Table S6. Scan speed on Exploris 480, Exploris 240, Exploris MX, and QE Plus instruments; Table S7. Complete data on the metabolite detected in full scan and SIM from a mouse-serum extract and from a mouse-liver extract (Excel table).
