# Supplementary material for: Selected Ion Monitoring for Orbitrap-Based Metabolomics
Source: Metabolites. 2024 Mar 25;14(4):184. doi: 10.3390/metabo14040184 (PMC11051813; doi:10.3390/metabo14040184)
Supplement: Supplementary file 1 [file metabolites-14-00184-s001.zip › metabolites-2895620 SM-proof.pdf]

Supplementary Materials

Selected Ion Monitoring for Orbitrap-Based Metabolomics

Wenyun Lu [1,2,3], Matthew J. McBride [1,4], Won Dong Lee [1], Xi Xing [1,2,3], Xincheng Xu [1,2], Xi Li [1,2,3], Anna M. Oschmann [1,2], Yihui Shen [1,3,5], Caroline Bartman [1,6] and Joshua D. Rabinowitz [1,2,3,7,8,*]

1 Lewis Sigler Institute for Integrative Genomics, Princeton University, Princeton, NJ 08544, USA; wlu@princeton.edu (W.L.); matthew.j.mcbride@rutgers.edu (M.J.M.); wdlee@princeton.edu (W.D.L.); xxing@princeton.edu (X.X.); xcxu@princeton.edu (X.X.); lixi@princeton.edu (X.L.); ao2098@princeton.edu (A.M.O.); yihuis@seas.upenn.edu (Y.S.); cbartman@pennmedicine.upenn.edu (C.B.)

2 Department of Chemistry, Princeton University, Princeton, NJ 08544, USA

3 DOE Center for Advanced Bioenergy and Bioproducts Innovation, Princeton University, Princeton, NJ 08544, USA

4 Department of Chemical Biology, Ernest Mario School of Pharmacy, Rutgers University, Piscataway, NJ 08854, USA

5 Department of Bioengineering, University of Pennsylvania, Philadelphia, PA 19104, USA

6 Department of Pharmacology, University of Pennsylvania, Philadelphia PA 19104, USA

7 Rutgers Cancer Institute of New Jersey (CINJ), Rutgers University, New Brunswick, NJ 08901, USA

8 Ludwig Institute for Cancer Research, Princeton University, Princeton, NJ 08544, USA

* Correspondence: Corresponding author: joshr@princeton.edu

Table of Contents

I. Supporting Methods…………………..…………………………………..……..S3-S8

S1. Chemicals and reagents

S2. Yeast culture condition

S3. Animal studies and tissue collection

S4. Animal infusions with ^{13}C-glucose

S5. [1-^{13}C]-2-deoxyglucose infusion and tissue collection

S6. [^{13}C]-formate infusion for mouse tumor

S7. Metabolite extraction from serum

S8. Liquid chromatography

S9. Using Xcalibur Qual Browser to obtain information on noise (N) and injection time (IT)

S10. Resolving power and scan speed on Exploris 480

S11. Natural isotope abundance calculation

S12. Setting up hybrid scan method covering both full scan and SIM on Exploris 480

II. Supporting Figures…………………..…………..……………………..………..S9-S21

Figure S1. Separation of selected metabolite isomers

Figure S2. Narrower scan width results in lower signal intensity

Figure S3. Inclusion of high intensity ions such as ^{12}C-glu diminishes the benefits of SIM

Figure S4. SIM does not improve S/N ratio for high intensity ions

Figure S5. Additional data for isotope ratio determination from mouse quadriceps extracts

Figure S6. Space charge caused signal drop and m/z shift for the glutamine ion from a liver extract when IT is long in SIM

Figure S7. Diagram showing the labeling positions of ATP from [^{13}C]-formate

Figure S8. Additional data on ATP labeling from [^{13}C]-formate infusion

Figure S9. Resolving power and scan speed on Exploris 480

Figure S10. The prominent peaks from phosphate at high concentrations from a T-cell extract

Figure S11. Detection of CoA metabolites using SIM vs. full scan from a mouse liver extract

Figure S12. The interference peak of $^{13}C_1$-2-DG6P from a mouse colon extract

Figure S13. Examples of interference peaks from in-source fragments

III. Supporting Tables……………………………………………………………..S21-S22

Table S1. List of ~600 metabolite standards with RT (Excel table)

Table S2. Additional data on SIM vs. full scan for six isotope labeled standards spiked into a mouse liver extract (Excel table)

Table S3. Additional data on the detection of ten low abundance metabolites from a mouse kidney extract, and from an I.o. extract (Excel table)

Table S4. Additional data on isotope ratio determination from mouse quadriceps extracts (Excel table)

Table S5. Signal intensity and mass accuracy of glutamine ion from a mouse liver extract in full scan (m/z 70-1000) or SIM (m/z 144.5-145.5) under different AGC target and IT_{max} settings (Excel table)

Table S6. Scan speed on Exploris 480, Exploris 240, Exploris MX and QE Plus instruments

Table S7. Complete data on the metabolite detected in full scan and SIM from a mouse serum extract, and from a mouse liver extract (Excel table)

I. Supporting methods

S1. Chemicals and reagents

HPLC-grade water (W6), acetonitrile (A955), and methanol (A456) were obtained from Thermo Fisher. Other components for the LC mobile phase are ammonium hydroxide (A669S-500, 28.0 to 30.0 w/w %, Fisher), and ammonium acetate (238074, ≥97%, Sigma). U-^{13}C-Glucose (CLM-1396, 99%) was obtained from Cambridge Isotope Laboratories. All other chemicals were obtained from Sigma. Metabolite standards were obtained from Sigma (St. Louis, MO), or Avanti (Alabaster, Al).

S2. Yeast culture condition

Wild type *I. orientalis* SD108 were cultivated in minimal media containing 6.7g/L yeast nitrogen base (YNB) without amino acids (Sigma-Aldrich, Y0626) and 20g/L glucose (Sigma-Aldrich, D9434). Media sterilization was carried out using 0.22 µm pore filters. *I. orientalis* colony was first inoculated into the minimal media to grow overnight in a shaker at 250 rpm and 30 °C until it reached an OD_{600} of 3. The overnight culture was inoculated into fresh media with an initial OD_{600} of ~0.1, and the culture was allowed to grow to exponential phase, and then metabolism quenched and metabolites extracted at OD_{600}=1.

S3. Animal studies and tissue collection

Animal studies followed protocols approved by the Princeton University Institutional Animal Care and Use Committee (IACUC). Three- to five-month-old male and female wild-type C57BL/6 mice (The Jackson Laboratory) were maintained on standard mouse chow, euthanized by cervical dislocation, and tissues quickly dissected and snap frozen in liquid nitrogen with a precooled Wollenberger clamp. This quenches metabolic activity and helps to prevent loss of unstable compounds. Frozen samples from liquid nitrogen were then transferred to −80°C freezer for storage. Blood was collected by tail snip (~10 µl) using blood collection tubes with clotting factor (Sarstedt 16.442.100).

S4. Animal infusions with ^{13}C-glucose

A tether and swivel system (Instech Laboratories, Plymouth Meeting, PA) was used for all infusions to provide free movement of the mouse in the cage. Mice were provided with bedding materials and access to food and hydrogel (Clear H2O, Portland, ME). For ^{13}C-glucose infusion, 400mM [U-^{13}C]glucose (Cambridge Isotope Laboratories, Tewksbury, MA) was infused through a jugular vein catheter connected to the mice, at a rate of 0.1 µl per minute per gram of body weight for 3 hours.

S5. [1-^{13}C]-2-deoxyglucose infusion and tissue collection

[1-^{13}C]-2-deoxyglucose (99% purity, CLM-1824, Cambridge Isotope Laboratories) was diluted to 8.3mM in sterile saline and stored at 4 ^{0}C. Jugular vein catheterized mice were fasted by switching to a fresh cage with no food at 9 am. At around 1:30 pm, mice were connected to the infusion line with swivel and tether (Instech products: swivel SMCLA, line KVABM1T/25) and infusion pump (syringepump.com, NE-1000), with infusate advanced through the tubing to the point of connection with the mouse. Before attaching line, each mouse was confirmed to have blood backflow through the jugular vein catheter, indicating that infused tracer would enter directly into the mouse bloodstream. The mice were weighed to calculate tracer infusion rate. Each mouse was left in a cage connected to a line for 1-2 hours to acclimate.

Between 2:30 and 3:30pm, infusion was started: first, the mouse was given a priming dose of 13 microliters (to clear mouse catheter up to jugular vein) in 30 seconds, and then the infusion rate was slowed to 0.3 microliters*(mouse weight in grams) per minute. After 10 minutes of infusion, the mice were euthanized quickly by cervical dislocation, the colon was dissected, colonic contents were quickly expelled, and tissue was freeze-clamped using a liquid nitrogen-cooled Wollenberger clamp. Tissues were stored at -80^{0}C until processed.

S6. [^{13}C]-formate infusion for mouse tumor

Male C57BL/6 mice from Charles River Laboratories were used at the age of 9 to 15 weeks. Mice were housed under a normal light cycle (7 am to 7 pm), with water and food (PicoLab Rodent Diet 5053, LabDiet) provided ad libitum. With sterile technique, catheters were inserted into the right jugular vein and mice were allowed to recover for at least 5 days. To initiate tumors, 5×10^5 MC38 cells (Kerafast) were injected subcutaneously with 100 μL of PBS on the left flank of each mouse. When tumors reached 100-400 mm^3, sodium formate (^{13}C, Cambridge Isotope Laboratories, CLM-583) dissolved in 0.9% sterile NaCl was infused at the rate of 8 and 30 nmol/min/g bodyweight (by volume 0.1 μL/min/g bodyweight) from 7 pm to 8 am of the next day. At the end of infusions, blood was collected by tail snip, and mice were euthanized by cervical dislocation. Tumors were dissected, wrapped in foils, clamped by a Wollenberger clamp precooled in liquid N_2, and stored at -80^{0}C until processed.

S7. Metabolite extraction from serum

For blood samples, blood was kept on ice for up to 60 min after sampling, then centrifuged at 4 ^{0}C 15000 RCF for 10 minutes. Serum fraction was transferred to another tube and stored at -80 ^{0}C. Serum samples were extracted in methanol at a ratio of 1:30 serum:methanol (v/v), centrifuged at 4 ^{0}C 15000 RCF for 20 minutes, then transferred to LCMS vials for measurement.

S8. Liquid chromatography

LC separation was achieved using a Waters XBridge BEH Amide column (2.1x150mm, 2.5 μm particle size, Part number 186006724), with column oven temperature at 25 °C and injection volume of 5 μL. The method has a running time of 25 minutes at a flow rate of 150 μL/min with a stepped gradient that provides good separation for a number of polar metabolite isomers. Solvent A is 95:5 water:acetonitrile with 20 mM ammonium hydroxide and 20 mM ammonium acetate, pH 9.4. Solvent B is acetonitrile. The gradient is, 0 min, 90% B; 2 min, 90% B; 3 min, 75%; 7 min, 75% B; 8 min, 70%, 9 min, 70% B; 10 min, 50% B; 12 min, 50% B; 13 min, 25% B; 14 min, 25% B; 16 min, 0% B, 20.5 min, 0% B; 21 min, 90% B; 25 min, 90% B. As seen in **Figure S1**, this method provides good separation for isomers of fructose, mannose, glucose and inositol, isomers of leucine and isoleucine, isomers of sarcosine, alanine and β-alanine, isomers of 2-hydroxybutyrate, 3-hydroxybutyrate, 3-hydroxyisobutyrate and gama-hydroxybutyrate, isomers of dimethylglycine, 2-aminobutyrate, 3-aminobutyrate and gamma-aminobutyrate. Using authentic standards, we determined the retention times (RT) of ~600 polar metabolites with this method (**Table S1**).

S9. Using Xcalibur Qual Browser to obtain information on noise (N) and injection time (IT)

Open the raw data file using Qual Browser. At the apex of the extracted ion chromatogram of the compound of interest, find the corresponding mass spectrum. Click "Display" on the main menu and click "Display Options". Under "Label", check "Noise". The noise (N) will be displayed on the mass spectrum as a number.

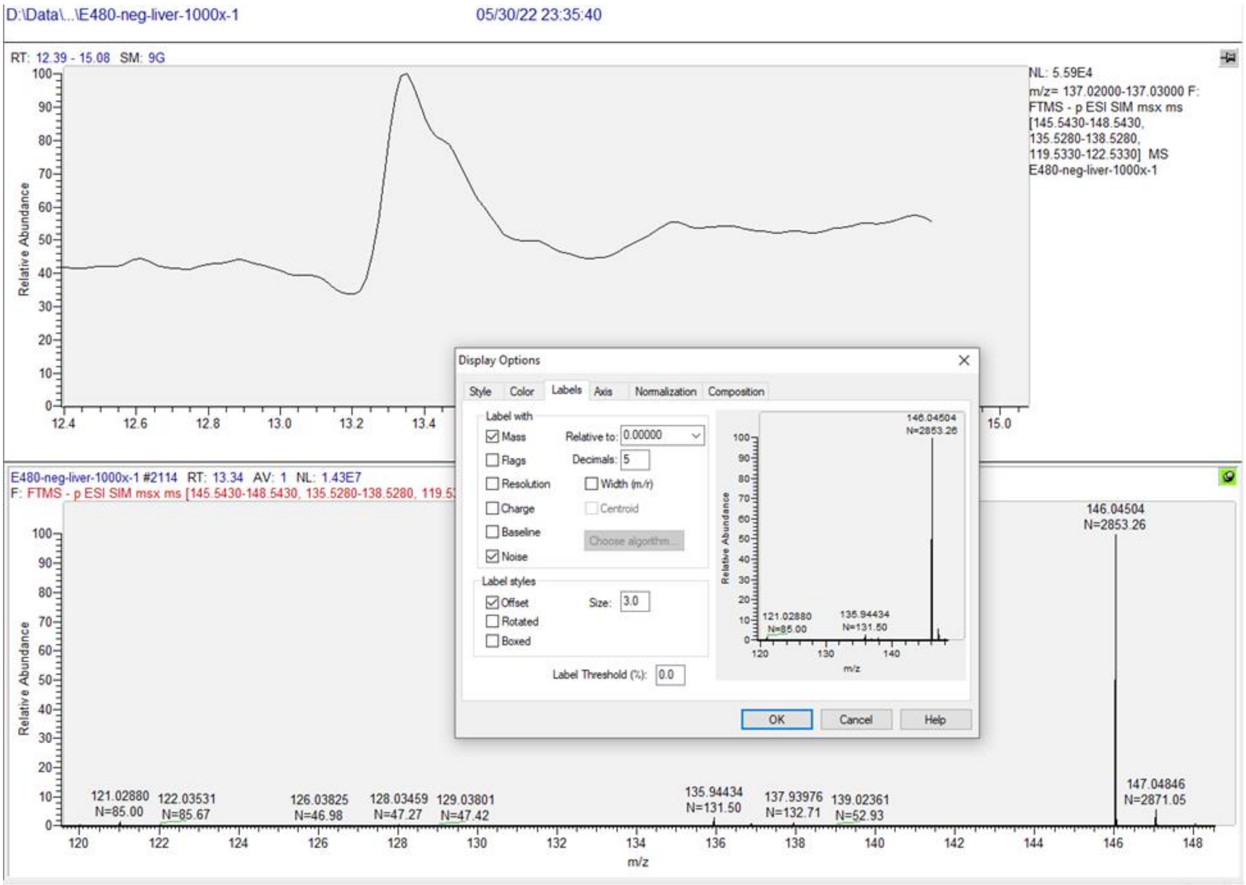

Using Xcalibur Qual Browser to obtain information on noise (N)

To find the injection time (IT), click “View” on main menu, and click “Scan Header”. This will bring up the scan header which contains information on IT. For the example shown below, there are three SIM scans in multiplex mode: *m/z* 145.54-148.54, *m/z* 135.53-138.53, and *m/z* 119.53-122.53. The injection times are 12 ms, 249 ms, and 339 ms, respectively. Note that in multiplex mode, the IT for individual SIM may exceed IT_{max} of 200 ms (as seen in the IT plot in **Figure 1C**), as long as the total IT of the three SIMs equals 200 * 3 = 600 ms.

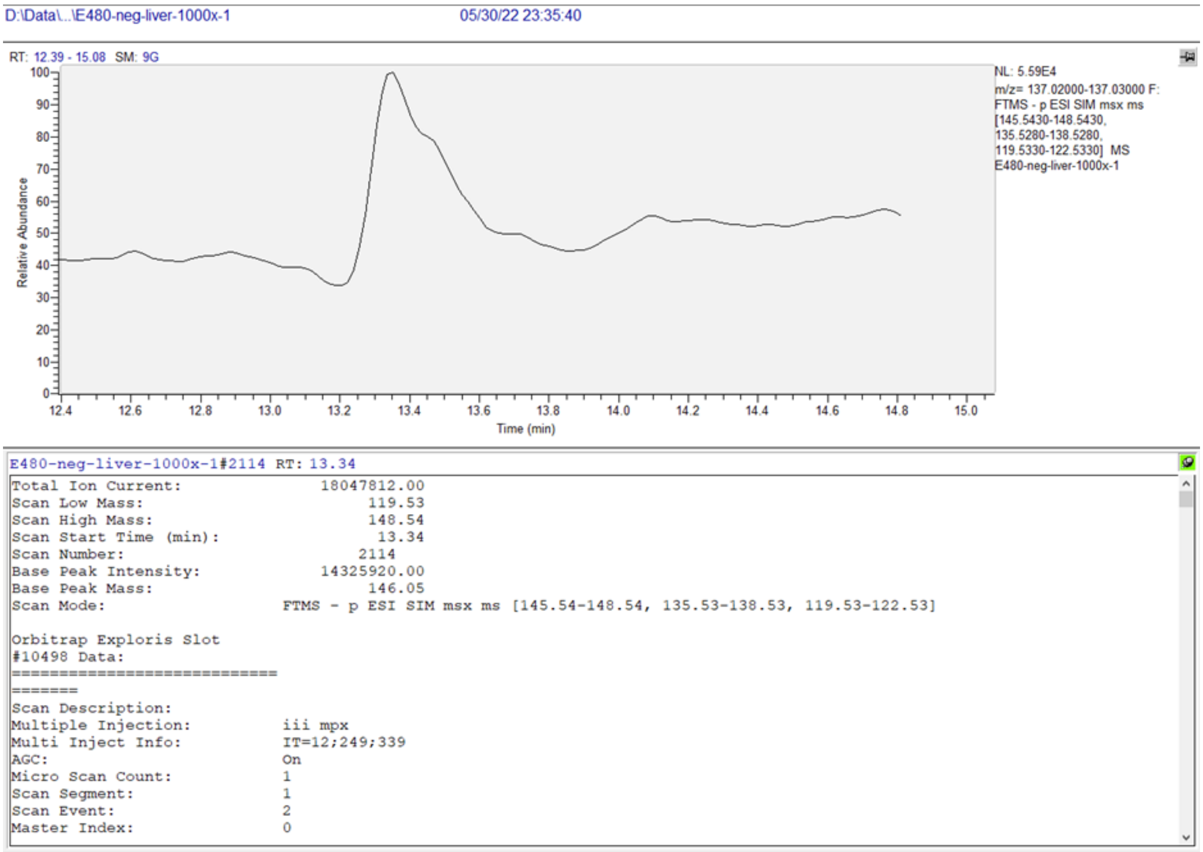

Using Xcalibur Qual Browser to obtain information on injection time (IT)

S10. Resolving power and scan speed for Exploris 480 orbitrap mass spectrometer

Thermo Orbitrap Exploris family offers four instruments that have different characteristic performance in terms of maximum resolving power at *m/z* 200 and MS1/MS2 capability: Exporis 480 (480K, MS1 and MS2), Exploris 240 (240K, MS1 and MS2), Exploris 120 (120K, MS1 and MS2), Exploris MX (180K, MS1 only). One key benefit of a high-resolution instrument is its ability to separate ions with similar masses, which is crucial for targeted or untargeted metabolomics, as well as isotope tracer-based flux studies (fluxomics). For example, protonated $^{13}C_2$-PC(38:6) (*m/z* 808.57611) and protonated PC(38:5) (*m/z* 808.58508) are not resolved on Q Exactive Plus (R=80K at *m/z* 800) and Exploris 240 (R=120K at *m/z* 800), and are fully resolved on Exploris 480 (R=240K at *m/z* 800) (**Figure S9**). Isotopic tracer studies bring additional isotopic labeled peaks in addition to the unlabeled peaks, making it even more necessary to use high resolving power to distinguish the target ions from interference ions. For example, we detected an interference peak near the mass of $^{13}C_1$-deoxyglucose 6-phosphate (*m/z* 244.0306 versus *m/z* 244.0310) in mouse colon samples when infusing with $^{13}C_1$-2-deoxy-D-glucose. The two peaks are poorly resolved on the Q Exactive Plus with a resoling power of 100K at *m/z 244*, and better resolved on Exploris 480 with a resolving power of 400K at *m/z* 244 (**Figure S12**). Note that the high resolving power alone is not sufficient to resolve interference peaks from in-source fragments that share the same *m/z* values as the target analytes (**Figure S13**). It is possible to distinguish these in-source fragments from the target analytes by chromatographic separation on column.

The high resolving power, on the other hand, comes at a price of slower scan speed. For Exploris 480, the average time between data points in negative mode is 0.35 seconds at 120K resolving power and 1.13 seconds at 480 K resolving power, respectively. It increases to 1.06 and 2.60 seconds in polarity switching mode (**Table S6**). Thus, it is important to choose appropriate resolving power and scan speed to have good chromatogram coverage. In practice, for routine metabolomics studies, we found that operating at

resolving power of 120K with polarity switching provides adequate chromatogram coverage with ~1 data point per second.

S11. Natural isotope abundance calculation

The $^{13}C_1$ and $^{18}O_1$ natural isotope abundances were calculated using Isotope Simulation program in Thermo Xcalibur Qual Browser software, assuming Gaussian peak profile.

Calculated natural isotope abundance

**Metabolite**	**Formula**	**$^{13}C_1$ (%)**	**$^{18}O_1$ (%)**	**Resolving power used for simulation***
3PG	$C_3H_7O_7P$	3.34	1.44	130K
Hexose-6P	$C_6H_{13}O_9P$	6.73	1.86	110K
FBP	$C_6H_{14}O_{12}P_2$	6.91	2.51	100K

*: The calculated isotope abundance will be affected by the resolving power used. The resolving power was chosen to match the experimental setting.

S12. Setting up hybrid scan method covering both full scan and SIM on Exploris 480

A mouse liver extract and a mouse serum extract were analyzed in full scan mode first. From the full scan data, low intensity metabolite ions were identified with information on *m/z* and RT. This information was used to set up a method that contains a full scan, as well as a multiplex SIM scan covering the targeted low intensity ions. Screenshots of the method setup are provided below. The parameters for full scan are as follows: resolution 60K, scan range 70-1000, RF lens 60%, AGC target 1e7, IT_{max} 100 ms. The parameters for SIM are as follows: maximum number of multiplexed ions 20, scan width 3.6 *m/z*, resolution 60K, AGC target 1e6, IT_{max} 30 ms, RT window 1.6 min.

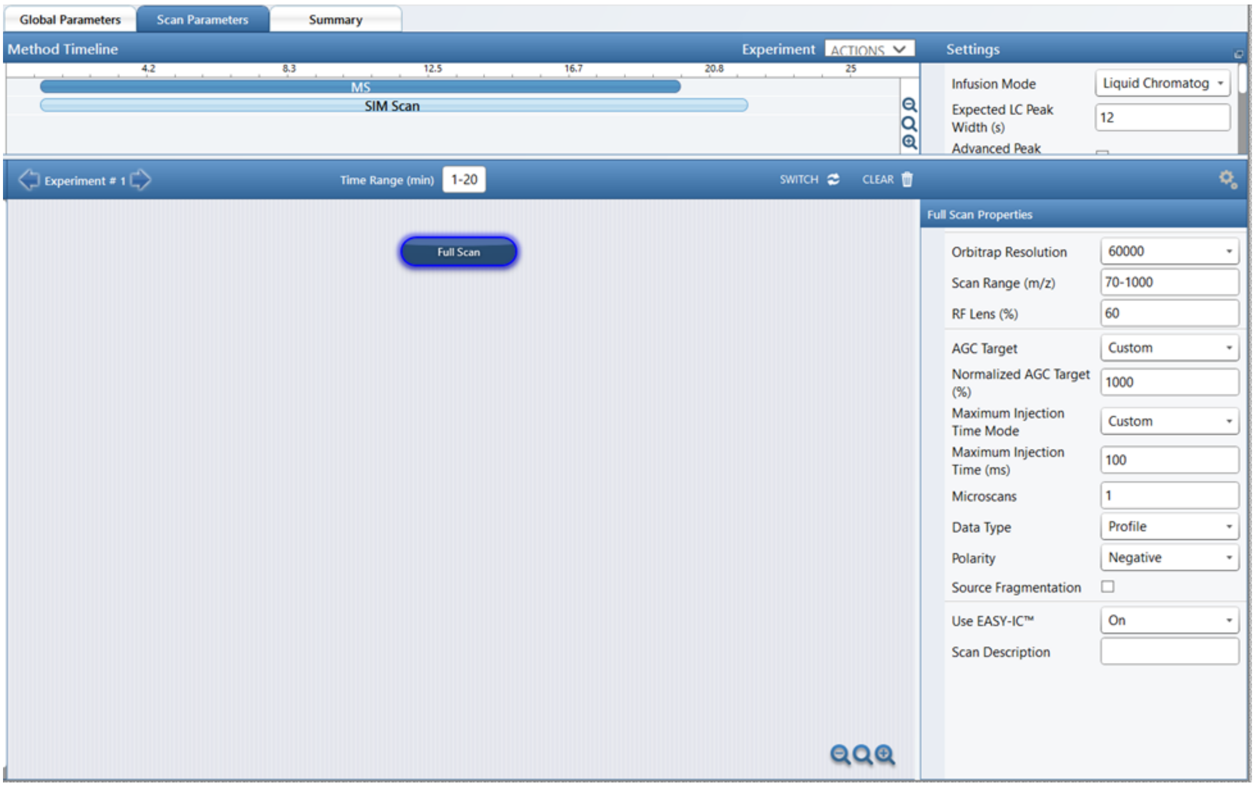

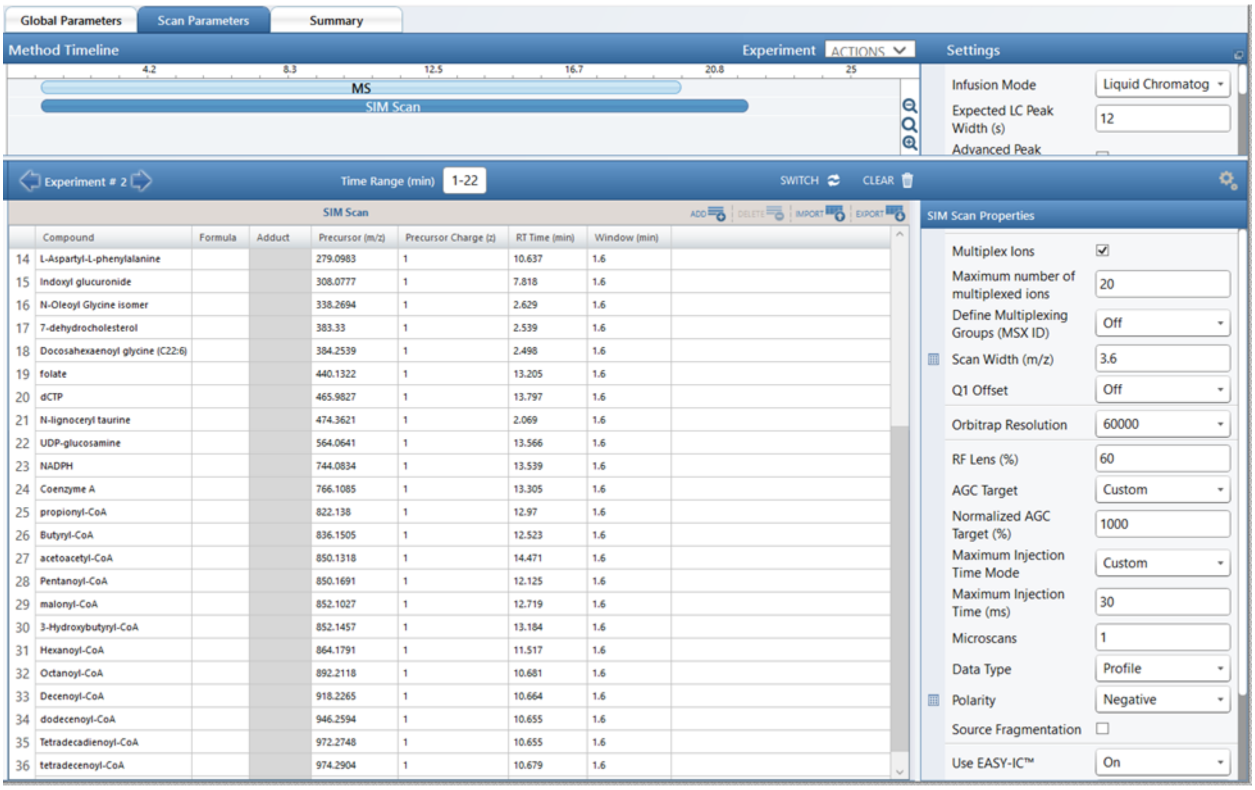

Screenshots showing the method setup

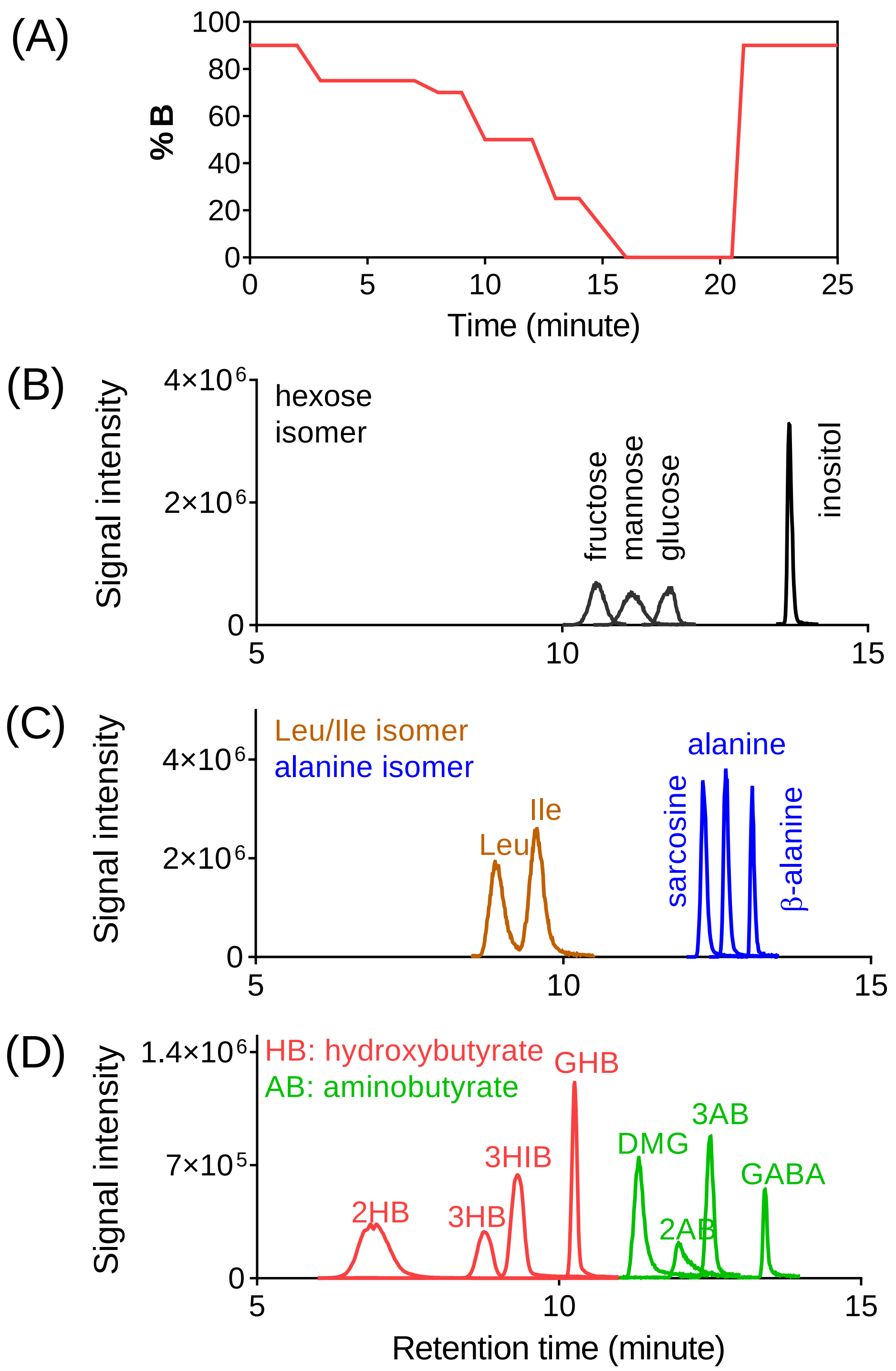

Figure S1. (A) The gradient for the 25-min HILIC method showing the percentage of solvent B (%B) as a function of time. (B) Chromatograms of standards of fructose, mannose, glucose, and inositol at 5 µg/mL. (C) Chromatograms of standards of leucine, isoleucine, sarcosine, alanine and β-alanine at 2 µg/mL. (D) Chromatograms of standards of 2-hydroxybutyrate (2HB), 3-hydroxybutyrate (3HB), 3-hydroxyisobutyrate (3HIB), and γ-hydroxybutyrate (GHB), dimethylglycine (DMG), 2-aminobutyrate (2AB), 3-aminobutyrate (3AB), and γ-aminobutyrate (GABA) at 1 µg/mL.

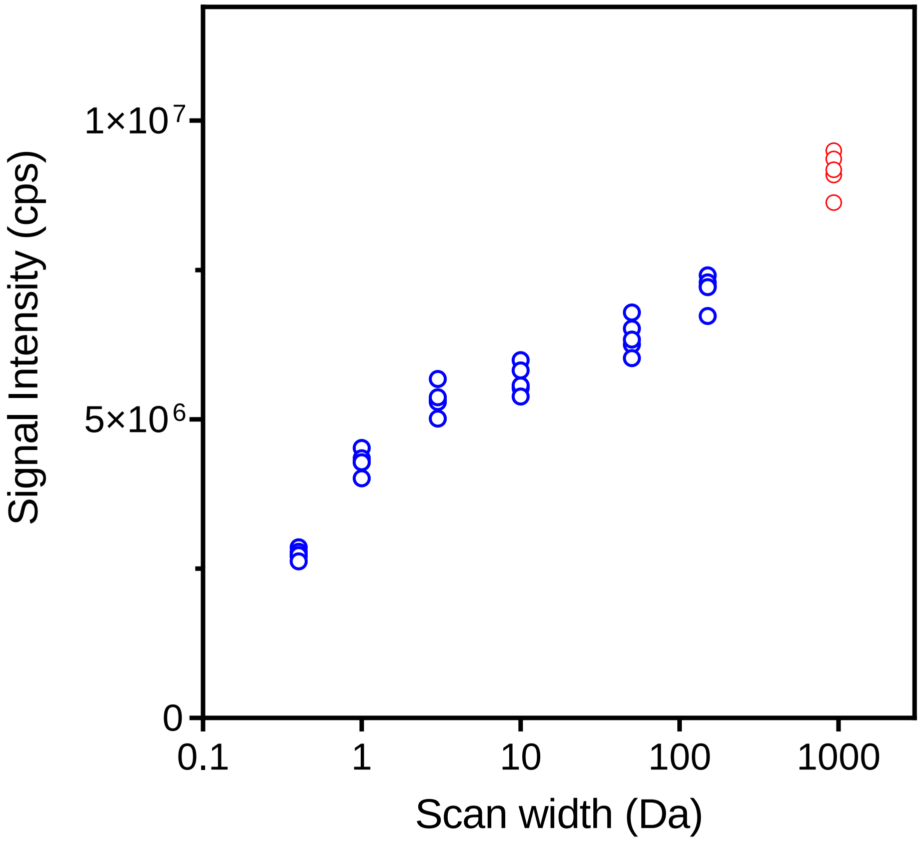

Figure S2. Signal intensity of tryptophan ion (*m/z* 203.0826) from a standard at 5 µg/mL detected in SIM mode with varying scan width (0.4, 1, 3, 10, 50, 150 Da, centered at *m/z* 203.08 in negative mode) and full scan mode (*m/z* 70-1000). Narrower scan width results in lower signal intensity. (N=5 technical replicates).

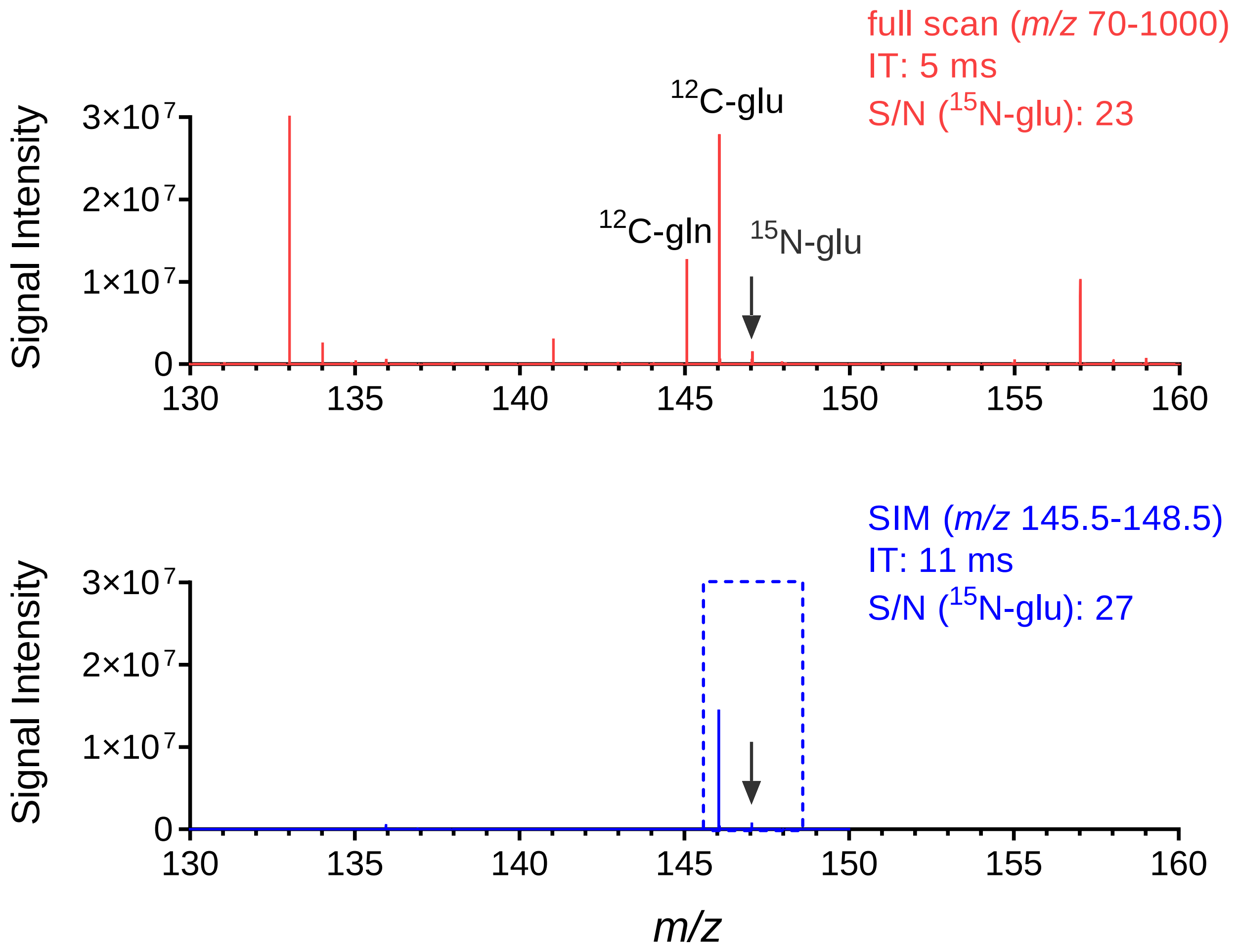

Figure S3. For the detection of ^{15}N-glu, S/N increases slightly from 23 in full scan to 27 in SIM due to the inclusion of the ^{12}C-glu which is of high abundance. In contrast, for the other five standards shown in **Figure 1D**, S/N increases >4-fold from full scan to SIM.

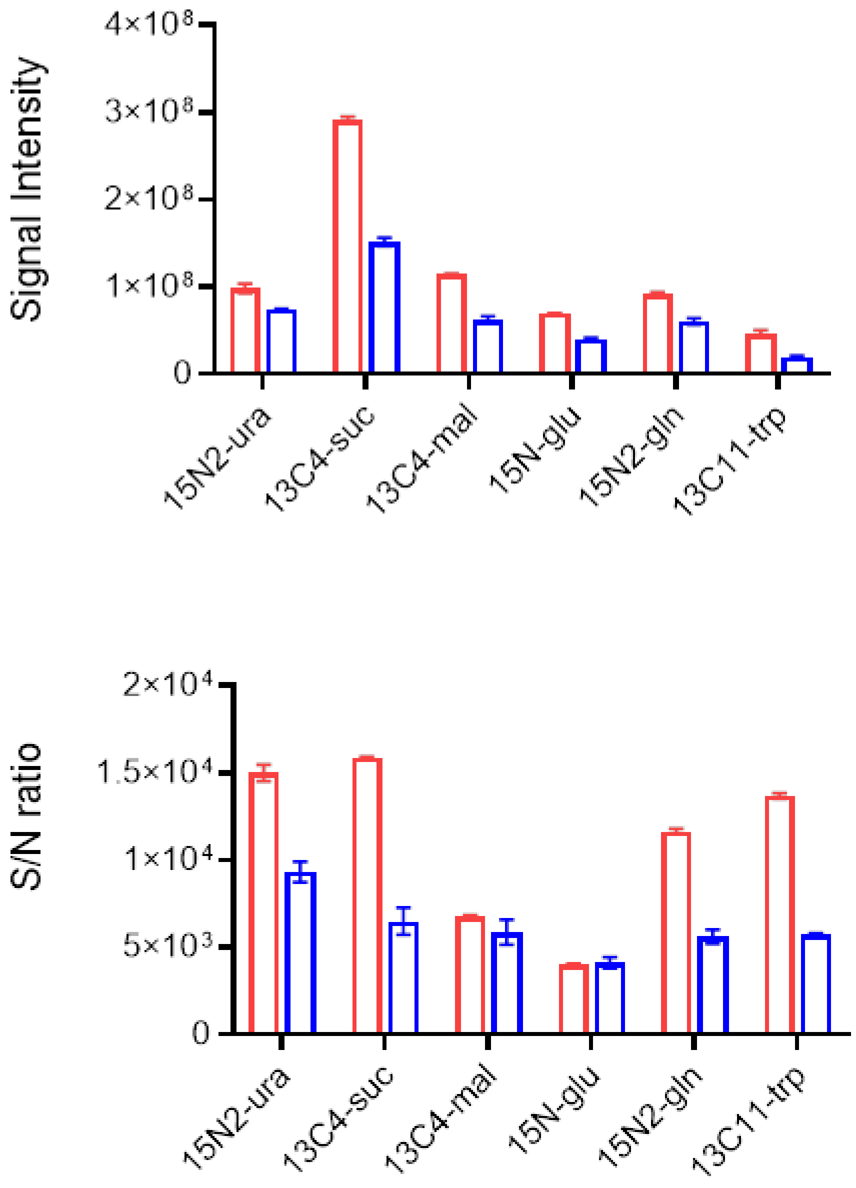

Figure S4. SIM does not improve S/N ratio for high intensity ions. For the six standards at high concentrations, signal/noise (S/N) ratios are lower in the SIM mode (blue bar) comparing to that in full scan mode (red bar). (**Table S2**).

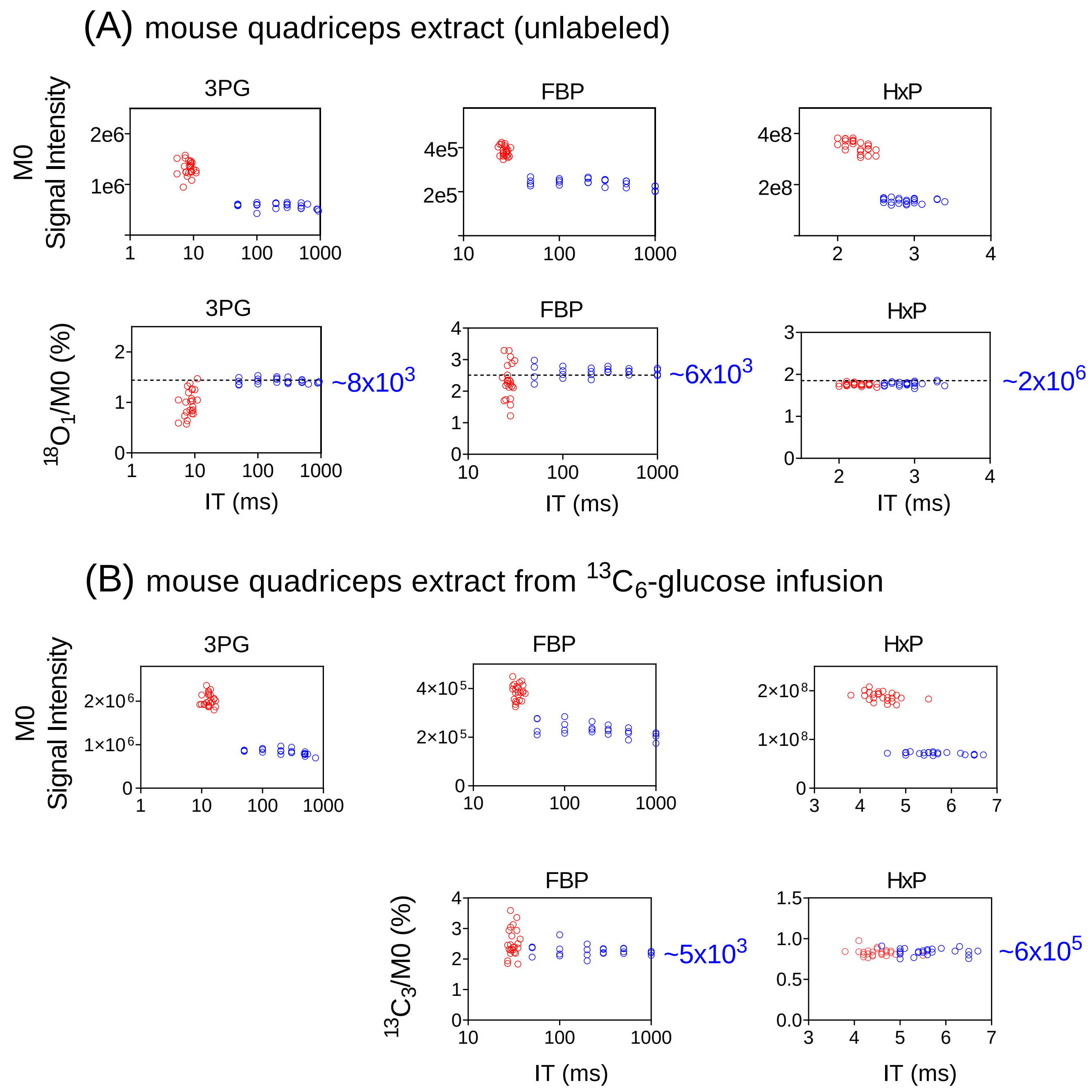

Figure S5. Additional data showing SIM improves isotope ratio determination for low abundance ions. (A) Upper panel: signal intensity for M0 peak of 3PG, FBP and Hexose-6P at various injection time (IT) for full scan (red dots) or SIM (blue dots) from an unlabeled mouse quadriceps extract. Lower panel: The ratio of ^{18}O peak over M0 peak for 3PG, FBP and hexose-6P as a function of IT; approximate signal intensity for ^{18}O peak is shown on the right for SIM. SIM improves the ^{18}O isotope ratio determination when absolute intensity is low (e.g., FBP and 3PG). (B) Upper panel: signal intensity for M0 peak of 3PG, FBP and Hexose-6P at various injection time (IT) for full scan (red dots) or SIM (blue dots) from a mouse quadriceps extract from $^{13}C_6$-glucose infusion. Lower panel: The ratio of $^{13}C_3$ peak over M0 peak for FBP, and hexose-6P as a function of IT; approximate signal intensity for $^{13}C_3$ peak is shown on the right for SIM. SIM improves the $^{13}C_3$ isotope ratio determination when absolute intensity is low (e.g., FBP).

(A)

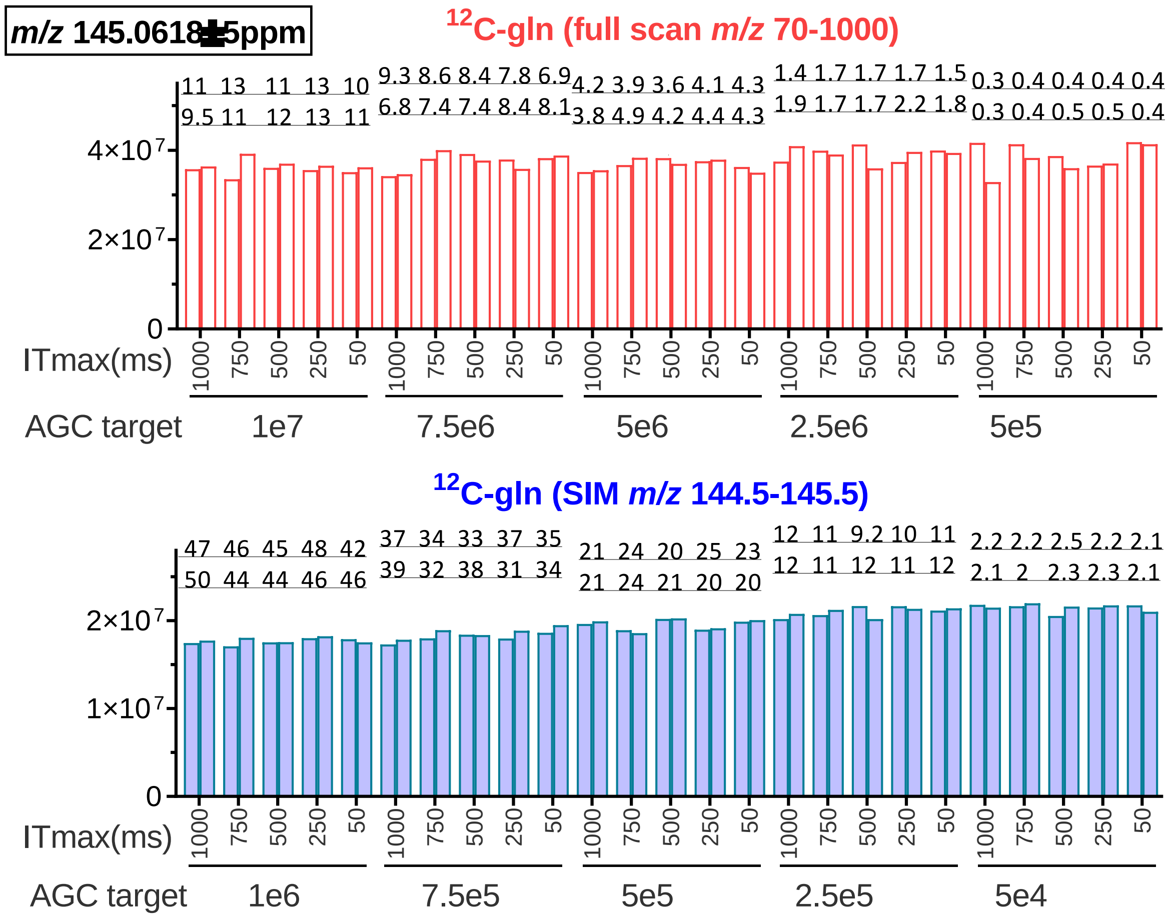

(B)

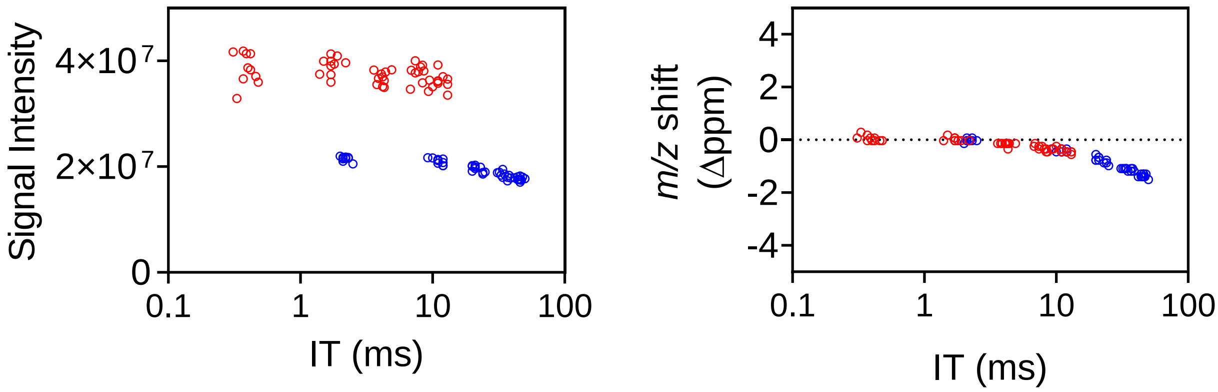

Figure S6. (A) Signal intensity of glutamine (*m/z* 145.0618±5ppm in negative mode) from a liver extract in full scan (*m/z* 70-1000, red) or SIM (144.5-145.5, blue) under different AGC target and IT_{max} settings. The actual IT is shown on top of the bar graph. Signal is consistent when IT is in the range of 0.3-10 ms for both full scan and SIM. Signal decreases when IT increases (> 10 ms), especially when IT reaches ~50 ms in SIM, due to space charge effect. (B) The signal drop at longer IT is accompanied by *m/z* shift. Full data is in **Table S5**.

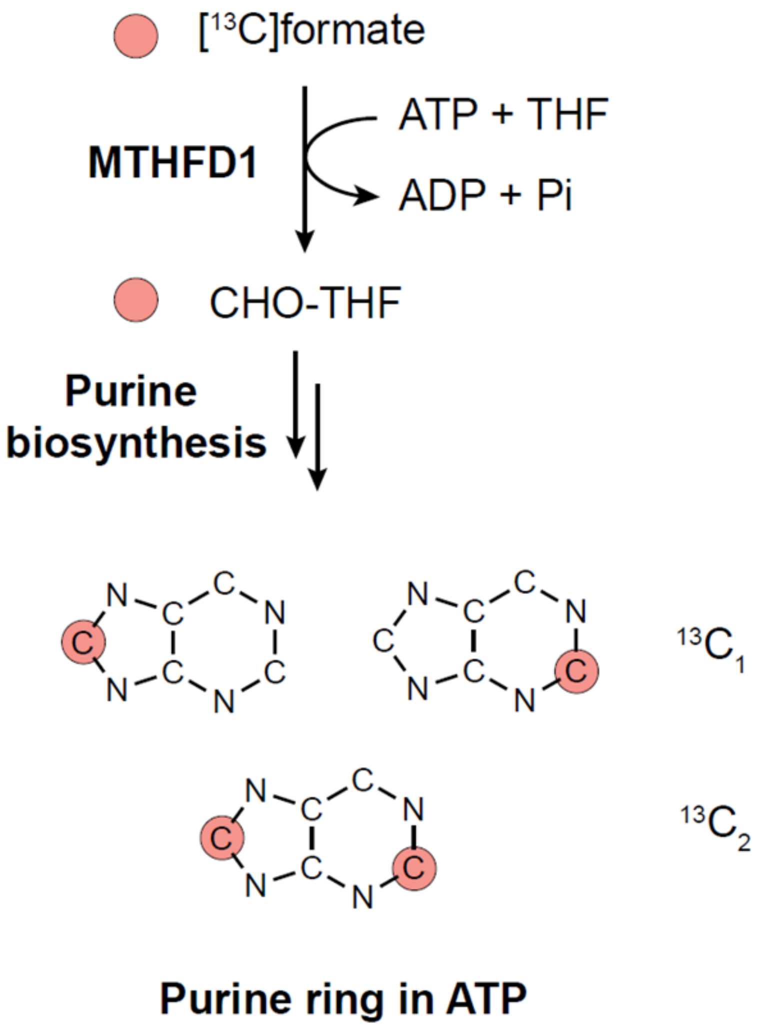

Figure S7. Diagram showing the labeling positions of ATP from [^{13}C]-formate tracing.

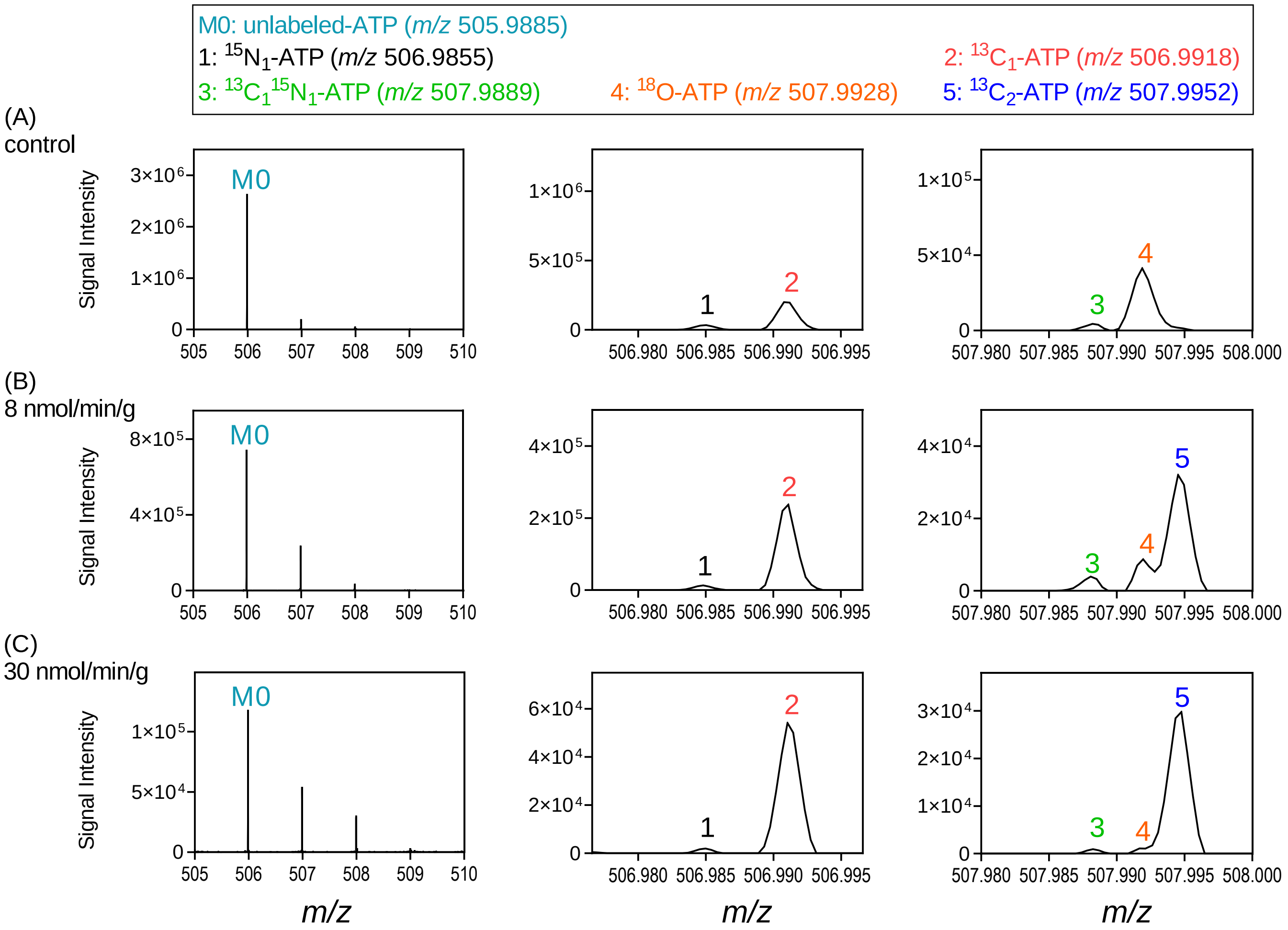

Figure S8. SIM scans using low AGC target and/or IT_{max} setting allows the reliable detection of labeling of ATP from [^{13}C]-formate infusion at various infusion rates: no infusion control (A), 8 nmol/min/g body weight (B) and 30 nmol/min/g body weight (C). The ions of interest are $^{13}C_1$-ATP (peak 2), and $^{13}C_2$-ATP (peak 5) which are resolved from other isotope peaks using the following settings: scan range *m/z* 504-510 in negative mode, resolving power 480K, AGC target 2e4, IT_{max} 50 ms. Higher infusion rate results in increased labeling of ATP.

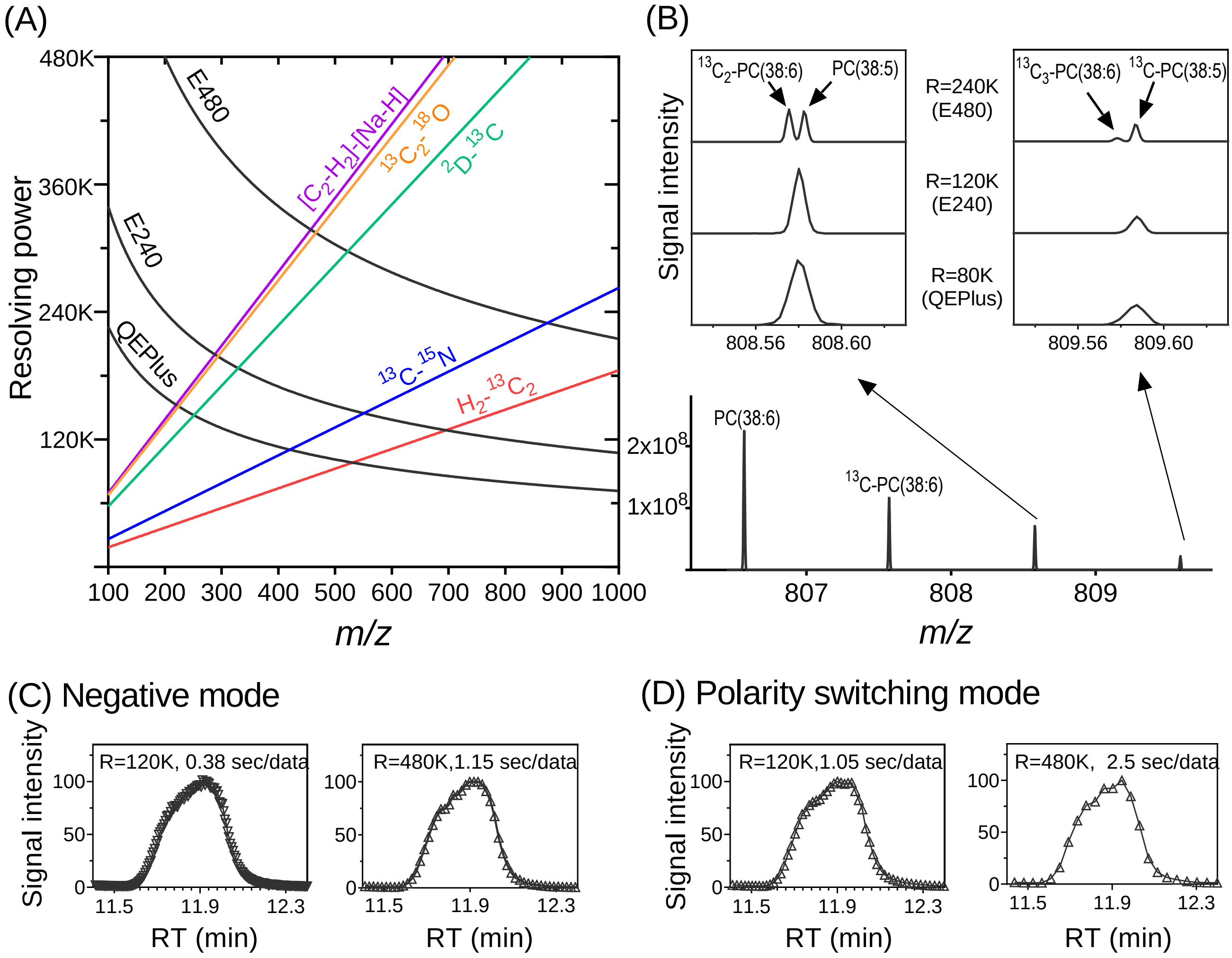

Figure S9. (A) The maximum resolving power of the three Orbitrap instruments (Q Exactive Plus, Exploris 240, Exploris 480), and the resolving power needed to separate five pairs of ions plotted as a function of *m/z*. The resolving power needed to separate two ions with mass difference of Δm is calculated as m/Δm*1.66 [1]. The five pairs of ions plotted are: (1) H_2-$^{13}C_2$: 0.00897 Da (for example, $^{13}C_2$-PC38:6+H$^+$ ($^{13}C_2{}^{12}C_{44}H_{80}NO_8P$+H$^+$) at *m/z* 808.57611, and PC38:5+H$^+$ ($C_{46}H_{82}NO_8P$+H$^+$) at *m/z* 808.58508 in positive mode); (2) ^{13}C-^{15}N: 0.00632 Da (for example, ^{15}N-glutamine at *m/z* 146.05890 and ^{13}C-glutamine at 146.06522 in negative mode); (3) ^{2}D-^{13}C: 0.00292 Da (for example, ^{13}C-glutamine at *m/z* 146.06522 and ^{2}D-glutamine at *m/z* 146.06814 in negative mode); (4) $^{13}C_2$-^{18}O: 0.00246 Da (for example, ^{18}O-glutamine at *m/z* 147.06611 and $^{13}C_2$-glutamine at *m/z* 147.06857 in negative mode); (5) [C_2-H_2]-[Na-H]: 0.00244 Da (for example, PC38:5+Na$^+$ ($C_{46}H_{82}NO_8P$+Na$^+$) at *m/z* 830.56702, and PC40:8+H$^+$ ($C_{48}H_{80}NO_8P$+H$^+$) at *m/z* 830.56946 in positive mode). (B) The high resolving power of Exploris 480 allows the separation of protonated $^{13}C_2$-PC(38:6) (*m/z* 808.57611) and PC(38:5) (*m/z* 808.58508), as well as protonated $^{13}C_3$-PC(38:6) (*m/z* 809.57947) and ^{13}C-PC(38:5) (*m/z* 809.58844). The two pairs of ions are not resolved on Exploris 240 or Q Exactive Plus. (C, D) Comparing the scan speed of Exploris 480 at resolving power of 120K and 480K in negative mode (C) and polarity switching mode (D), for the detection of glucose anion (*m/z* 179.0561) in full scan mode from a serum extract. Higher resolving power results in slower scan speed. The scan range is *m/z* 70-1000 for negative mode with AGC target of 1e7 and IT_{max} of 300 ms. For polarity switching mode, there are two scan events, *m/z* 70-1000 in negative mode and *m/z* 120-1000 in positive mode.

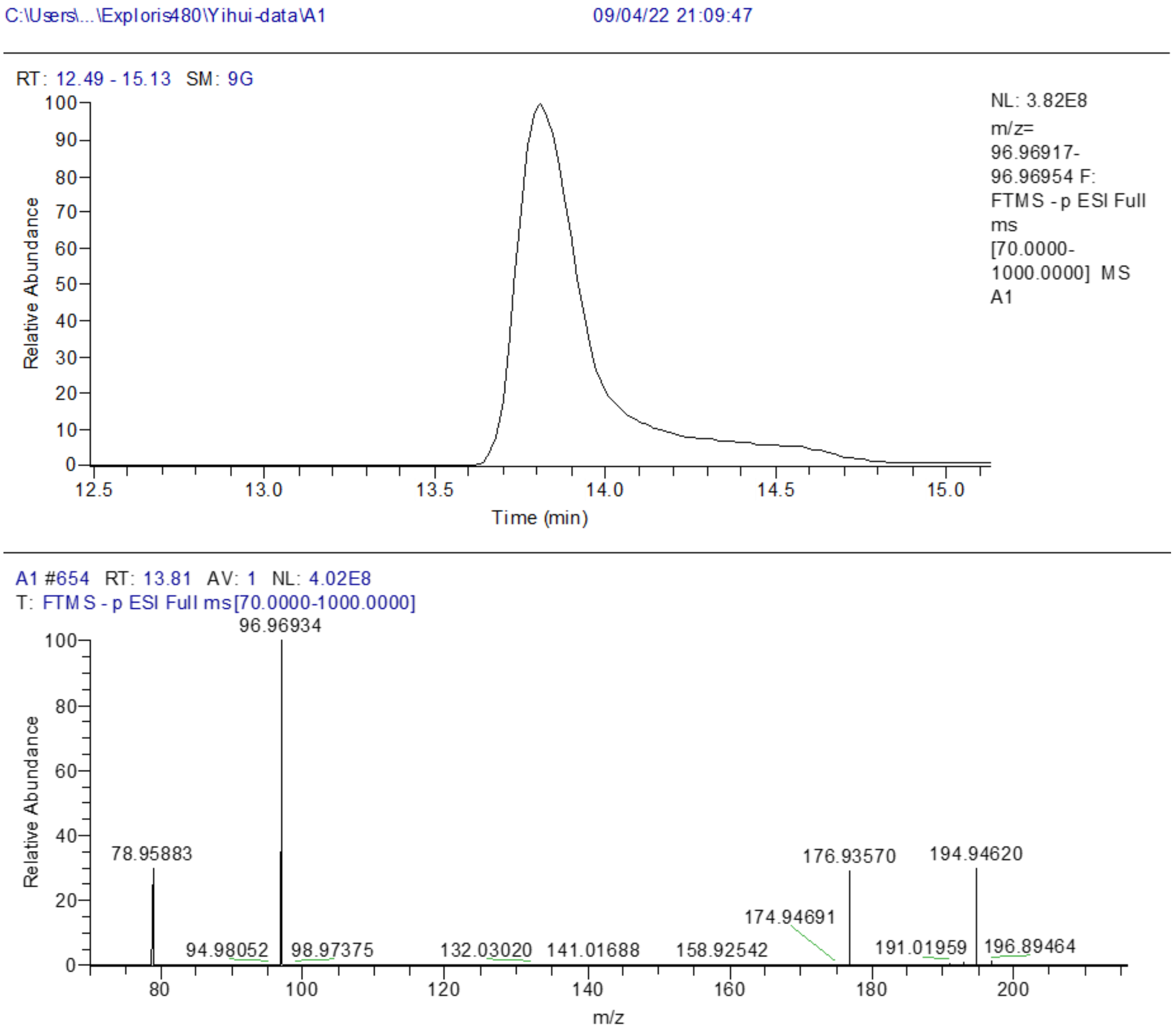

Figure S10. The presence of phosphate at high concentration in a T-cell extract results in prominent peaks at *m*/*z* 78.96, 96.97, 176.94, and 194.95 in negative mode.

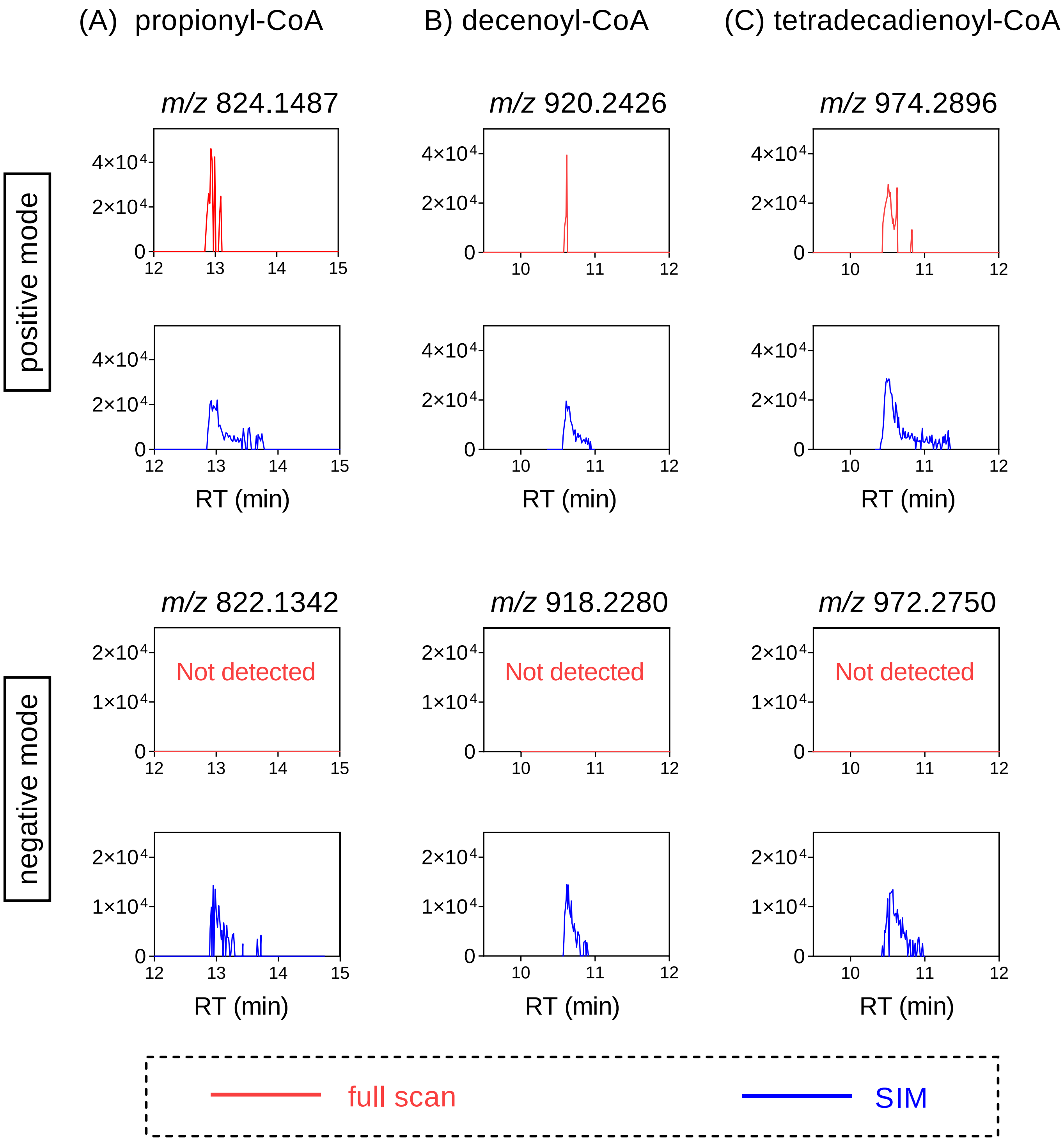

Figure S11. Extracted ion chromatograms for three CoA species detected in full scan (red trace) and SIM (blue trace), respectively, from a mouse liver extract. (A) propionyl-CoA. (B) decenoyl-CoA. (C) tetradecadienoyl-CoA. In all cases, SIM provides more accurate quantitation.

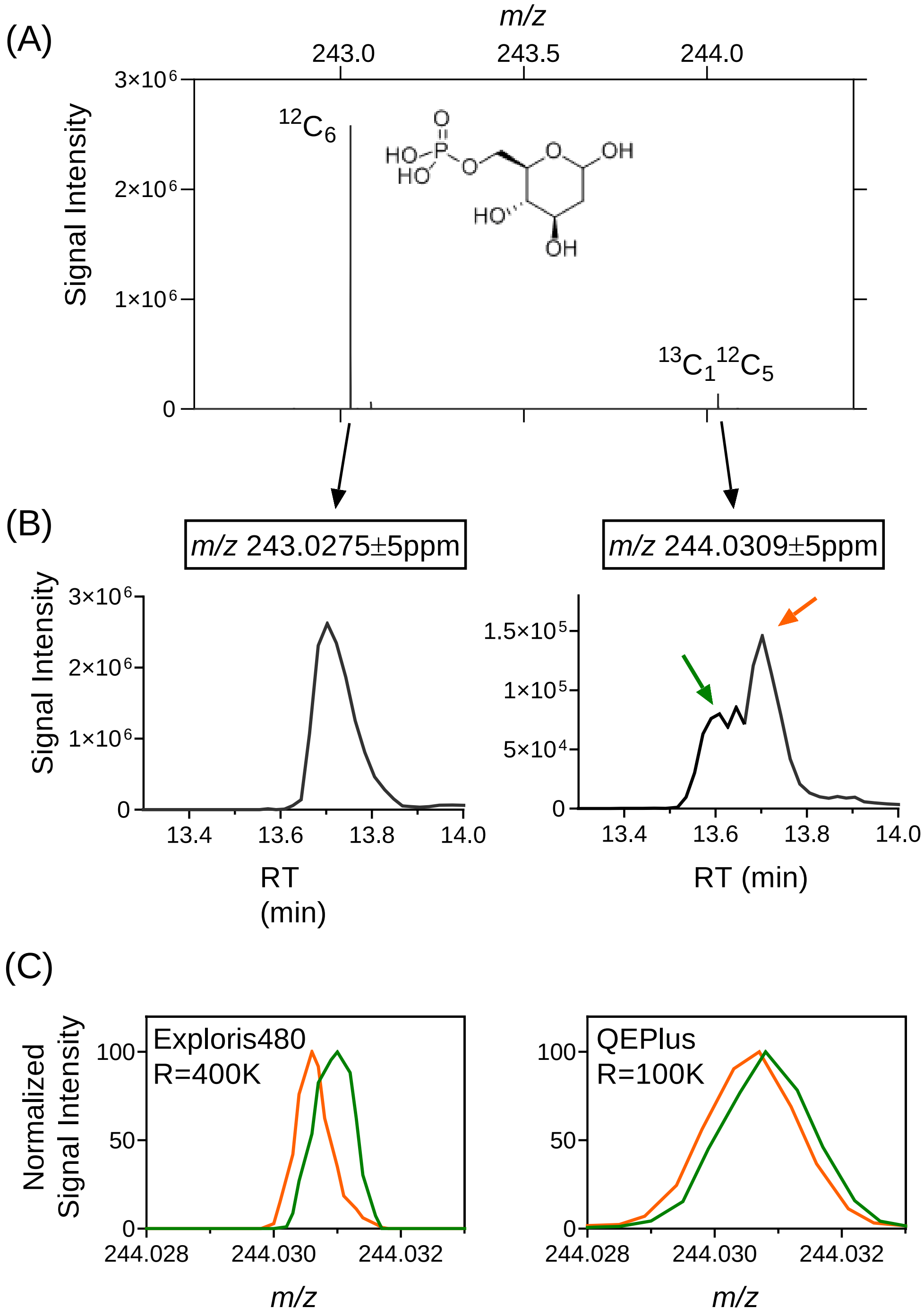

Figure S12. Mass spectrum at the retention time of 2-deoxy-D-glucose 6-phosphate (2-DG6P) (A) and the corresponding chromatograms of 2-DG6P (theoretical *m/z* of 243.0275, detected *m/z* of 243.0272) and $^{13}C_1$-2-DG6P (theoretical *m/z* of 244.0309, detected *m/z* of 244.0306) (B, orange arrow). There is an interference ion at similar retention time with detected *m/z* of 244.0310 (B, green arrow). The two ions are poorly resolved on the Q Exactive Plus instrument, and better resolved on the Exploris 480 instrument (C).

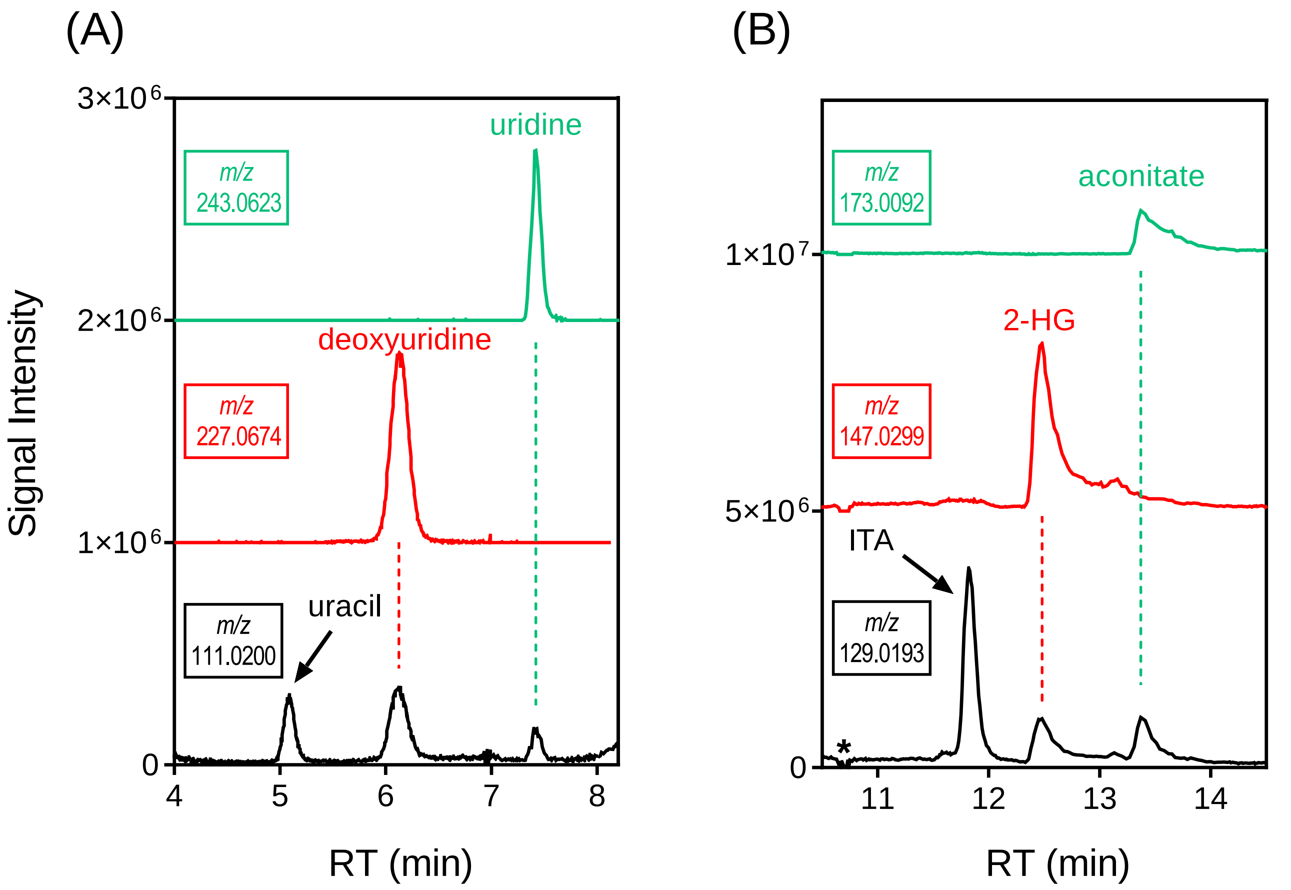

Figure S13. Examples of interference peaks from in-source fragments. (A) The extracted ion chromatogram of uracil in negative mode (*m/z* 111.0200±5ppm) from a mouse serum extract shows three peaks, uracil (RT 5.09 min), a fragment from deoxyuridine (RT 6.14 min), and a fragment from uridine (RT 7.42 min). (B) The extracted ion chromatogram of itaconate (ITA) in negative mode (*m/z* 129.0193±5ppm) from a mouse liver extract shows three peaks, ITA (RT 11.81 min), a fragment from 2-hydroxyglutarate (2-HG, RT 12.47 min), and a fragment from aconitate (RT 13.37 min). The apparent signal dip at ~10.70 min is due to ion suppression at the electrospray ionization source from co-eluting taurine with a signal intensity of ~$6x10^8$ (*).

Table S1. List of ~600 metabolite standards with information of retention time (RT) for the 25-min HILIC method (**Figure S1**). (Excel table).

Table S2. Comparing the injection time (IT, ms), normalized signal intensity (S, ion counts per second), electronic noise (N), S/N ratio of full scan versus SIM, for six isotope labeled standards spiked into a mouse liver extract, at two concentrations that are of 1000-fold difference. Data was taken at the apex of the corresponding chromatogram. Data are plotted in **Figure 1C, 1D and Figure S4**. (N=2 technical replicates) (Excel table).

Table S3. Data on the signal intensity of ten low abundance metabolites from a mouse kidney extract, and from an *I. o.* extract for full scan and SIM. Samples were run five times to obtain RSD with results presented in **Figure 2**. (Excel table).

Table S4. Data on the isotope ratios for various isotope forms for 3PG, HxP and FBP from a mouse quadriceps extract without or with $^{13}C_6$-glucose infusion under full scan or SIM with different AGC target and IT_{max} settings. Data are plotted in **Figure 3** and **Figure S5**. (Excel table).

Table S5. Signal intensity and mass accuracy of glutamine ion from a mouse liver extract in full scan (*m/z* 70-1000) or SIM (*m/z* 144.5-145.5) under different AGC target and IT_{max} settings. Data are plotted in **Figure S6**. (Excel table).

Table S6. Average time (in the unit of seconds) between data points from retention time of 1 to 18 min under various resolution settings (at *m/z* 200) on four instruments, from a liver extract (red) and a serum extract (green), respectively. The scan range is *m/z* 70-1000 for negative mode, while for polarity switching mode there are two scan events, one for *m/z* 70-1000 in negative mode and one for *m/z* 120-1000 in positive mode. For all scan events, AGC target is 1e7 for Exploris 480, Exploris 240, Exploris MX, and 5e6 for Q Exactive Plus, while IT_{max} is 300ms.

**Exploris 480**	***R=120K***	***R=240K***	***R=480K***
Negative mode	0.35, 0.37	0.61, 0.64	1.13, 1.16
Polarity switching mode	1.06, 1.10	1.57, 1.62	2.60, 2.66
**Exploris 240**	***R=60K***	***R=120K***	***R=240K***
Negative mode	0.38, 0.40	0.54, 0.58	0.80, 0.83
Polarity switching mode	1.03, 1.08	1.31, 1.36	1.83, 1.88
**Exploris MX**	***R=60K***	***R=120K***	***R=180K***
Negative mode	0.37, 0.38	0.45, 0.50	0.59, 0.64
Polarity switching mode	1.06, 1.06	1.26, 1.32	1.53, 1.60
**Q Exactive Plus**	***R=35K***	***R=70K***	***R=140K***
Negative mode	0.22, 0.25	0.35, 0.38	0.61, 0.63
Polarity switching mode	1.00, 1.10	1.26, 1.35	1.75, 1.84

Table S7. Signal intensity of metabolites detected in full scan and targeted SIMs in the same LC-MS run from a mouse serum extract, and a mouse liver extract, in positive mode and negative mode, respectively. Data are plotted in **Figure 5**. (N=5 technical replicates) (Excel table).

Reference (for figure legend of Figure S9).

(1) Su, X.; Lu, W.; Rabinowitz, J. D. Metabolite Spectral Accuracy on Orbitraps. *Anal. Chem.* **2017**, *89*, 5940-5948.
